# Occasional long-distance dispersal may not prevent inbreeding in a threatened butterfly

**DOI:** 10.1186/s12862-021-01953-z

**Published:** 2021-12-27

**Authors:** Annelore De Ro, An Vanden Broeck, Leen Verschaeve, Ilf Jacobs, Filiep T’Jollyn, Hans Van Dyck, Dirk Maes

**Affiliations:** 1 Research Institute for Nature and Forest (INBO), Gaverstraat 4, B-9500 Geraardsbergen, Belgium; 2 Natuurpunt Studie, Coxiestraat 11, B-2800 Mechelen, Belgium; 3 Research Institute for Nature and Forest (INBO), Herman Teirlinckgebouw, Havenlaan 88 box 73, B-1000 Brussels, Belgium; 4 Behavioural Ecology and Conservation Group, Biodiversity Research Centre, Earth and Life Institute, Université Catholique de Louvain (UCLouvain), Croix du Sud 4-5, B-1348 Louvain-la-Neuve, Belgium

**Keywords:** Butterfly conservation, Conservation units, Translocation, Effective population size, Gene flow

## Abstract

**Background:**

To set up successful conservation measures, detailed knowledge on the dispersal and colonization capacities of the focal species and connectivity between populations is of high relevance. We developed species-specific nuclear microsatellite molecular markers for the grayling (*Hipparchia semele*), a butterfly endemic to Europe and of growing conservation concern in North-West Europe, and report on its population genetics, in a fragmented, anthropogenic landscape in Belgium. Our study included samples from 23 different locations nested in two regions and additional historical samples from two locations. We assessed contemporary, long-distance dispersal based on genetic assignment tests and investigated the effect of habitat loss and fragmentation on the population genetic structure and genetic variation using data of nine microsatellite loci.

**Results:**

Detected dispersal events covered remarkably long distances, which were up to ten times larger than previously reported colonisation distances, with the longest movement recorded in this study even exceeding 100 km. However, observed frequencies of long-distance dispersal were low. Our results point to the consequences of the strong population decline of the last decades, with evidence of inbreeding for several of the recently sampled populations and low estimates of effective population sizes (*Ne*) (ranging from 20 to 54 individuals).

**Conclusions:**

Our study shows low frequencies of long-distance dispersal, which is unable to prevent inbreeding in most of the local populations. We discuss the significance for species conservation including future translocation events and discuss appropriate conservation strategies to maintain viable grayling (meta) populations in highly fragmented, anthropogenic landscapes.

**Supplementary Information:**

The online version contains supplementary material available at 10.1186/s12862-021-01953-z.

## Background

In recent years, terrestrial insect declines have been reported worldwide, with habitat loss and fragmentation as main drivers of population decline in anthropogenic landscapes [1]. Habitat loss and fragmentation result in increased distances between local populations and between such populations and vacant habitat patches. In such spatially heterogeneous environments, movement through the matrix typically comes with higher costs than movements within habitat patches [2]. A longer search time for resource patches in the landscape matrix will increase the cost of dispersal (e.g. mortality risks, predation risks, energetic reserve exhaustion, and deferred costs), which may further reduce the physiological conditions of immigrants; all these factors will contribute to reduced fitness of immigrants after they dispersed through the hostile matrix [3]. Habitat fragmentation can thus hinder or even prevent dispersal, and thus gene flow between populations. Furthermore, it can prevent natural colonisation when distances between populations or to potentially suitable habitat patches become greater than the dispersal ability of the species [4]. Subsequently, severely reduced gene flow and the effects of genetic drift can cause a loss of genetic diversity and increase genetic differentiation between historically connected populations, independent of local adaptation. Complete isolation of populations, among other factors such as population size, may eventually lead to an increase in inbreeding and extinction probability [5].

The grayling (*Hipparchia semele*) is a butterfly species endemic to Europe that is highly affected by habitat loss and fragmentation, in particular in NW Europe. It is listed as ‘Threatened’ in Flanders (northern Belgium) [6] and as ‘Vulnerable’ both in the UK [7] and the Netherlands [8]. In most NW European countries, the grayling mainly occurs in coastal dunes and inland heathlands which are fragmented and restricted to typically small and isolated remnants [9, 10] embedded in an anthropogenic and agricultural landscape matrix. In Belgium, for example, coastal dune areas decreased by 36% during the twentieth century mainly due to building for tourism [11]. Similarly, heathlands strongly decreased in northern Belgium; only 11% of the heathland surface was left in 1995 compared to 1965 and the decline is still ongoing [12]. Heathlands are negatively impacted by eutrophication through aerial nitrogen deposition, and the abandonment of traditional land use, such as sheep grazing and burning [9, 13]. European heathlands and coastal dunes with herbaceous vegetation ('grey dunes') are of high conservation priority and they are protected under the EU Habitats Directive [14]. The grayling can be considered as an umbrella species since it requires rather large patches of open vegetation of different succession stages, which offers habitat opportunities for several other invertebrates of European heathlands and coastal dunes [15].

For the conservation of species in fragmented biotopes that need additional measures relative to standard vegetation management, species action plans should address the coordination of conservation and management actions on a landscape-scale, encompassing a network of suitable habitats and metapopulations [16]. Conservation efforts should thus not only concentrate on maximizing the habitat quality of currently occupied patches, but also of vacant potentially suitable habitat which could be (re-)colonized [17] and on the improvement of connectivity between local populations. This helps species to move around the landscape and increase the rate of colonization and gene flow [18]. Clearly defined spatial conservation units are a handy tool for guiding conservation actions on a local scale. For the delimitation of such conservation units, detailed knowledge on the dispersal and colonization capacities of the focal species are required.

In demographic studies, dispersal is often estimated with direct methods such as mark-release-recapture (MRR) studies, the dynamics of patch colonization and patch extinctions or data on range expansions. Other mobility indices of insect species, such as mobility assessment (e.g. very sedentary versus highly mobile) and relative flight speed, are usually based on expert judgements [19]. These methods are often constrained by manpower and sampling area [20]. This makes rare long-distance movements difficult to detect within such studies, which may lead to the underestimation of the dispersal ability of species (e.g. [21]). Conversely, genetic methods provide indirect estimates of realized dispersal. Assignment tests based on molecular markers, among other indirect genetic methods, provide an alternative and powerful approach for getting more insight in the functional connectivity and gene flow between populations and long-distance movements [22].

While previous studies on the grayling focussed on habitat use and mobility [15, 23, 24], metapopulation dynamics [15, 25], disturbance tolerance [26] and local adaptation to climate variables [27], this study addressed the species’ potential for gene flow and dispersal ability based on neutral molecular markers. In this study, our aim was to (1) estimate contemporary, long-distance dispersal of the grayling in northern Belgium based on genetic assignment tests and (2) investigate how habitat loss and fragmentation affect the population genetic structure and patterns of genetic variation. We hereto developed species-specific polymorphic nuclear microsatellite markers, which will be useful tools for other population genetic studies of the grayling. Finally, we recommend appropriate conservation, translocation and reintroduction strategies to restore viable populations of the grayling in northern Belgium, a region characterized by highly fragmented landscapes under strong anthropogenic pressure.

## Methods

### Study species

Within Europe, the grayling *Hipparchia semele* (Linneaus, 1758) has a widespread distribution, but it is absent in the northernmost parts and the highest European mountains [28]. See Additional file 1: S1 for a map of the distribution of the grayling in North-West Europe. In Belgium, its occurrence is largely restricted to northern Belgium (Flanders), as most of the grayling populations in southern Belgium (Wallonia) have gone extinct [29]. Due to heathland fragmentation and the loss of heathland and grey dune dynamics, the species has suffered a decline in its distribution in northern Belgium of 47% since 1991 [30], 55% in the Netherlands since 1950 [8] and 62% in the UK since 1970 [7]. The grayling is a long-living butterfly species; adult individuals have a lifespan of up to 40 or even 60 days [31]. The species has one generation per year. The eggs hatch around August, and larvae grow in four instars from August to the following June.

### Study sites

**Fig. 1:**
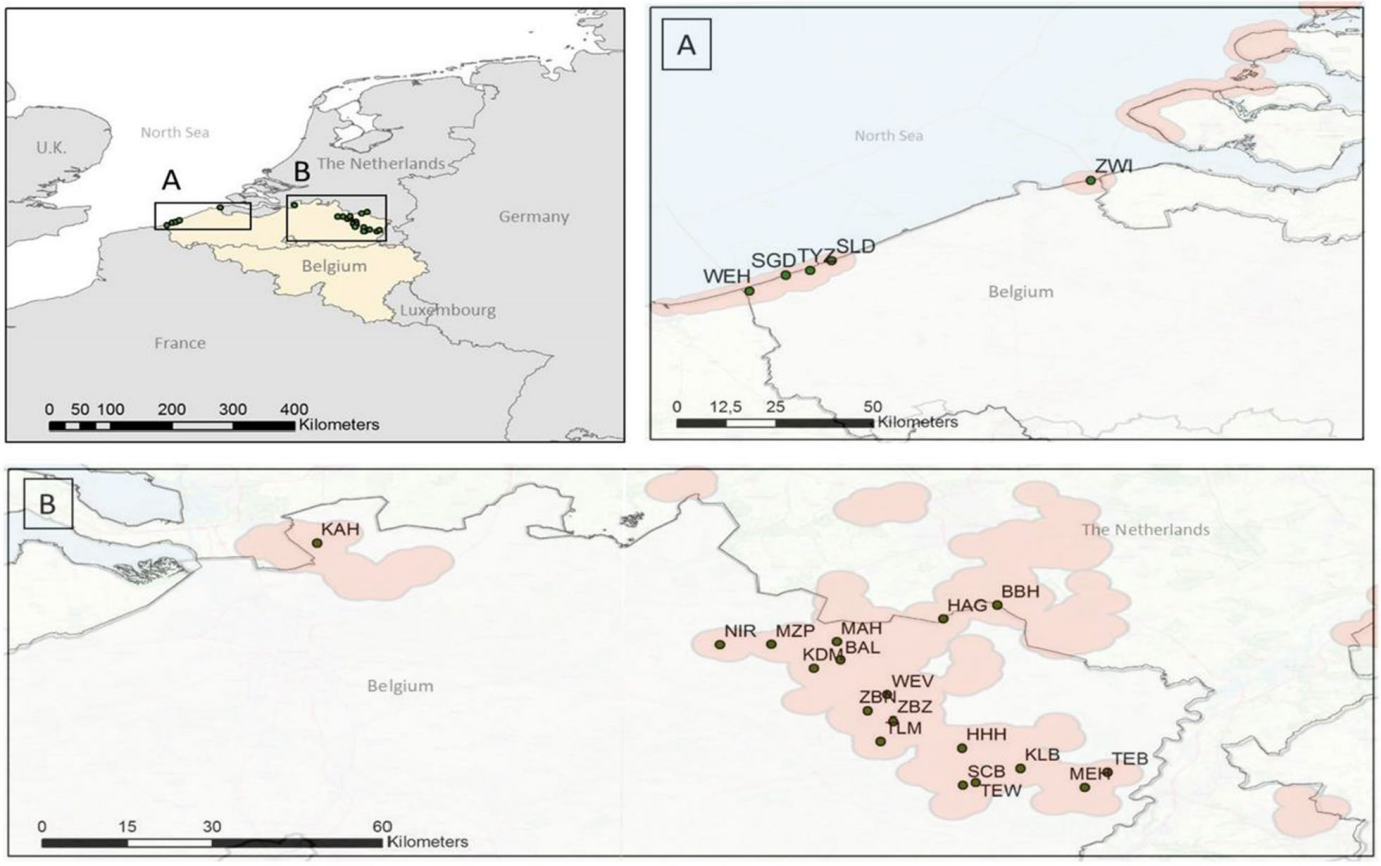
Sampling locations of *Hipparchia semele****.*** The sampling locations are situated in the coastal region **A** and the inland heathland region **B** of northern Belgium. Observations of *H. semele* since 2011 are clustered (orange polygons) with a 2.5 km radius. Sources: GBIF, iNaturalist and observado.

Current populations of the grayling were located by mapping observations made by skilled volunteers using an online data portal (http://waarnemingen.be). For our genetic study, we sampled the locations in northern Belgium where a minimum of ten individuals were observed in one year in the time period 2015–2019. Sampling occurred during the summer of 2020. We assumed that each sampling location contained a single deme; a local population consisting of closely related individuals that form a distinct gene pool, and where mating is random. We sampled 23 different locations nested in two different regions: the Belgian coast in north-west Belgium (five locations) and the inland Campine region located in the north-east of Belgium (18 locations) (Fig. 1, Table 1) (Additional file 1: S2). On two other inland locations that met our criteria (KSV, GRS), were no individuals found. The sampling areas ranged from 4197m^2^ to 487 435m^2^ with a mean of 76 865m^2^. The maximum distance between samples within a location ranged from 89 to 3132 m with a mean of 668 m. The distance to the nearest population ranged from 644 m to 46 730 m with a mean of 6800 m. Although we sampled all known recent populations in Belgium, we cannot exclude that we have missed a few recently colonized small populations within the regions.

**Table 1:** List of sampling locations, associated population genetic statistics and effective population sizes

Region	Location	Location Code	Mean_N	Ar	He	Inbreeding (F_IS_)	Ne (RM)	95% CI	
								L	U
Coast	Schipgatduinen	SGD	28.44	2.88	0.386	0.086	34	21	60
	Sint-Laureinsduinen	SLD	29.00	2.60	0.373	**0.278**	32	19	59
	Ter Yde—Zeebermduinen	TYZ	29.44	2.99	0.421	**0.155**	26	15	46
	Westhoek	WEH	29.33	2.92	0.411	**0.152**	44	26	85
	Zwin	ZWI	29.33	2.78	0.422	**0.138**	20	11	39
Inland	Balimgronden	BAL	28.11	3.26	0.419	**0.259**	30	18	57
	Beverbeekse Heide	BBH	26.00	3.38	0.413	**0.139**	33	19	60
	Hageven	HAG	26.89	3.22	0.440	**0.168**	35	21	63
	Kalmthoutse Heide	KAH	27.11	3.28	0.410	**0.159**	32	19	57
	Keiheuvel—De Most	KDM	25.33	3.40	0.394	**0.126**	37	22	66
	Klaverberg	KLB	21.78	3.07	0.399	**0.194**	36	20	72
	Mechelse Heide	MEH	18.78	2.90	0.372	**0.252**	33	18	66
	Militair Schietveld	HHH	27.44	3.26	0.407	0.065	36	22	64
	Molse Zandputten	MZP	28.56	2.98	0.422	**0.111**	21	12	40
	Niras	NIR	24.44	3.17	0.442	**0.195**	25	14	46
	Schemmersberg	SCB	27.89	3.04	0.350	− 0.001	25	14	46
	Terril Lindeman	TLM	27.22	3.61	0.462	**0.159**	32	18	58
	Terril Winterslag	TEW	22.78	3.45	0.381	**0.150**	30	17	57
	Teutelberg	TEB	19.89	2.91	0.372	0.142	32	18	64
	Weyersvlakte	WEV	28.89	3.51	0.428	**0.127**	35	21	66
	Zwarte Beek—Noord	ZBN	26.33	3.65	0.418	0.056	54	31	107
	Zwarte Beek—Zuid	ZBZ	23.89	3.35	0.398	0.054	39	23	84
	Mechelse Heide 2001	MEH	23.44	2.73	0.369	0.136	28	16	57
	Teutelberg 2001	TEB	16.89	3.17	0.396	0.101	31	16	69

The mean number of genotyped individuals per locus (*Mean_N*), allelic richness (*Ar*), expected heterozygosity (*He*), inbreeding coefficient *F*_*IS*_ (with the R package DiveRsity, populations with evidence for inbreeding are indicated in bold) and the estimated effective population size (*Ne*) of the sampled locations. *Ne* is estimated in the program Colony2 while using the Random Mating model (RM) together with a 95% confidence interval (95%CI, *L*  lower limit, *U*  upper limit)

### Sampling, DNA extraction and amplification

Per sampling location, we took wing-clips (2–3 mm^2^ tissue) of 30 grayling butterflies, except for the location Maatheide (MAH) and Teutelberg (TEB), where only 2 and 25 individuals were detected, respectively. This resulted in a total of 657 non-destructive wing-clip samples. Wing-clip sampling does not reduce flight ability nor does it affect survival rate (e.g.[32]), although there may be some short-term behavioural changes associated with capture and manipulating individuals [33]. Additionally, we included 24 and 20 grayling individuals collected in 2001 at the locations Mechelse Heide (MEH) and Teutelberg (TEB), respectively. They were stored at −80 °C. These historical samples allowed a comparison of genetic diversity at two time points of the latter populations. The wing-clips were preserved in cryotubes filled with 96% ethanol until analysis.

Before extraction of genomic DNA (Additional file 1: S3), each wing fragment was air-dried for one hour to vaporise all the 96% ethanol. Genomic DNA was extracted from each wing fragment, which was homogenized in a solution of 100 µL 6% Chelex InstaGene Matrix solution (Biorad) and 5 µL proteinase K (> 600 mAU/ml, Qiagen). Microsatellite development and genotyping services were carried out by AllGenetics & Biology SL (www.allgenetics.eu) (Additional file 1: S4). For each sample, 20 polymorphic nuclear microsatellites were amplified. PCR products were run on an ABI 3500 analyser with the GeneScan-600 LIZ size standard (Applied Biosystems) and analysed using Geneious Prime 2019.3.2 (https://www.geneious.com). Details on microsatellites and PCR conditions are given in Additional file 1: S5. Assessment of the genotyping error was performed by including duplicate DNA-extractions for 24 wing-clip samples and, in addition, two to five independent duplicated PCR-amplifications of 21 wing-clip samples.

### Data analysis

In total, 701 butterfly wing clips were genotyped. Five microsatellite loci were discarded from the analyses due to stutter peaks and subsequent difficulty in allele scoring. Hence, we worked with 15 microsatellite loci. Next, we examined the assumptions of Hardy–Weinberg equilibrium and of no linkage disequilibrium (LD). We used GENEPOP v4.3 [34] to test for significance of deviations from Hardy–Weinberg equilibrium at individual loci, corrected for multiple testing by the Bonferroni-method [35]. Next we tested for LD for each pair of loci in each population (using the Markov chain method and default parameter settings) and estimated the frequency of null alleles (r) by using the Dempster method [36] (Additional file 1: S6). A high amount of null alleles is common in butterfly studies (e.g. [37]), which we also found in this study. To check the influence of loci with deviation from Hard-Weinberg proportions and null alleles on further analyses on the robustness of the results, we followed Waples [38]. We therefore compared results for overall (*G’*_*ST*_*, D*_*EST*_) and population-specific genetic diversity (*F*_*IS*_*, Ho, He, Ar*), with and without each of these loci. Six loci were discarded during these steps and the two samples of location Maatheide (MAH) were discarded due to missing data at more than 3 loci. The following data analyses were performed based on nine microsatellite loci and 641 unique samples from 24 sampling locations (two and 22 locations sampled in 2001 and 2020, respectively). A total of 63 alleles, with an average of 3.6 alleles per locus, were observed and the dataset had an overall proportion of missing data of 3.7%. For additional information, see Additional file 1: S7.

#### Long-distance dispersal

Genetic assignment methods of dispersal inference make use of individual genotypes and population allele frequencies to estimate where individuals were born or not [39]. GENECLASS2 [40] uses individual assignment tests to determine the putative first-generation dispersers. We used the Frequency based method (Additional file 1: S8) and the Bayesian method as assignment criterion, of which the latter is assumed to be the most efficient method [41]. We did not sample populations in the neighboring countries (the Netherlands, Germany and France) and we cannot exclude that we have missed a few recently colonized small locations in Flanders. We therefore selected the L_home criterion (with L_home being the likelihood of drawing the individual genotype from the population where the individual has been sampled), which is recommended when it is known that not all populations have been sampled [40]. This criterion is however less powerful than the L_home/L_max criterion (the ratio of L_home to the maximum likelihood observed for drawing the individual genotype from any population, including where the individual was sampled) [39]. We ran 1000 Monte Carlo resampling algorithms. Covered distances of long-distance movements were measured as the distance between the location where the individual was assigned to (L_home) and the location where the individual was sampled.

#### Genetic diversity

For each sampled location, we calculated the average number of individuals genotyped per locus (*N*), average number of observed alleles per locus (*A*), effective number of alleles (*Ae*), average observed heterozygosity (*Ho*), Nei’s overall expected heterozygosity (*He*) and the mean inbreeding coefficient (*F*_*IS*_) using GenAlEx v6.501 [42] and the R package DiveRsity [43]. We additionally calculated F_IS_ corrected for the potential presence of null alleles with the program INEst 2.2 [44]. Next, we used the rarefaction method implemented in HP-Rare v1.0 [45] to calculate the average number of alleles per locus (*Ar*) and number of private alleles (*Ap)* adjusted for sample size, based on a minimum sample size of 13 individuals. To detect significant differences in genetic diversity indices, ANOVA analysis were conducted in R [46]. We tested for recent bottlenecks using the program BOTTLENECK v1.2.02 [47] which evaluates deviations of *He* from the values expected at mutation–drift equilibrium (*He* > *He eq*) (Additional file 1: S9). We estimated the contemporary, local effective number of individuals (*Ne*) for each sampling location by using the linkage disequilibrium method (LDNe) implemented in the program NeEstimator v2 [48] and the sibship assignment method in the program Colony2 [49, 50] (Additional file 1: S10). The correlation of the area of the site and the distance to the nearest population to *Ne*, *F*_*IS*_, *Ar* and *He* were tested with univariate linear regressions in R [46].

#### Population differentiation

We used the R package DiveRsity to calculate global, regional and pairwise genetic differentiation between populations. We calculated G’_ST_ [51] and additionally D_EST_ [52] based on 1000 bootstraps. Both G’_ST_ and D_EST_ are zero (or slightly negative) when there is no differentiation between populations and one at complete differentiation. Differences in the distribution of genotypes between all population pairs were assessed through calculation of 95% confidence intervals (C.I.) of the G’_ST_ values with a bias-corrected bootstrapping method (1000 bootstraps). Using the ISOLDE program in GENEPOP v4.3, we tested for an overall and regional pattern of genetic heterogeneity over geographic distances with the isolation-by-distance-test (IBD) [34]. We used linearized F_ST_-estimates (F_ST_/(1–F_ST_)) for pairs of populations and the Euclidian geographic distance in kilometres between sampling locations. We set the number of permutations for Mantel test to 100 000 and the rest of the parameters on default parameter settings. Next, we used GenAlEx v6.501 to estimate levels of hierarchical structuring within populations, among populations and among regions by analysis of molecular variance (AMOVA) with populations nested within two regions (coastal and inland heathland) (999 permutations). Bayesian analyses of population structure was performed with the programs STRUCTURE v2.3.4 [53] and BAPS v6 [54] (Additional file 1: S11). Lastly, we further investigated the genetic structure among populations using a Principal Component Analysis (PCA) in the program GenAlEx v6.501.

## Results

### Long-distance dispersal

**Fig. 2:**
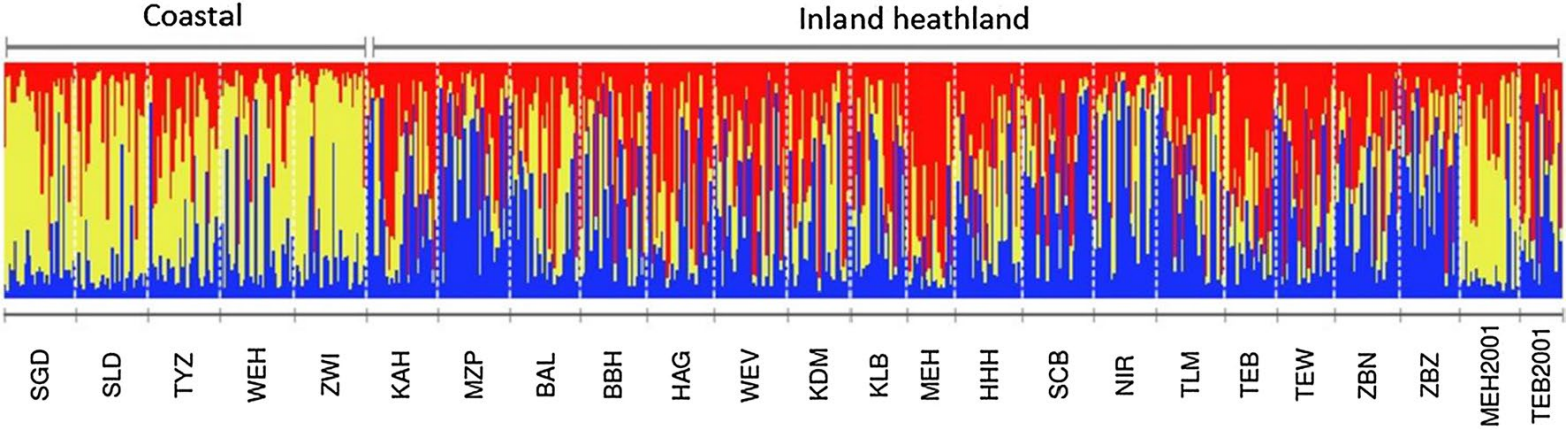
STRUCTURE Bayesian clustering of the sampled *Hipparchia semele* individuals. The optimal number of clusters K was 3. Each column represents an individual and is divided according to its probability of membership of different clusters, which are represented by different colours. The region to which the populations belong is indicated above the columns

The population assignment test using the Bayesian method identified seven putative first-generation long-distance dispersers (p ≤ 0.01) (Table 2). Putative dispersal events mainly occurred between inland populations (range: 13–69 km, mean: 33 km). Remarkably, one putative disperser originated in a coastal population (ZWI) and moved to an inland population (TLM), covering a distance of 142 km. This putative long-distance dispersal event was also detected by the STRUCTURE analysis (Fig. 2) where the latter putative disperser was assigned to the coastal population cluster. An extra quality and scoring check of the genetic profiles of this putative long-distance disperser confirmed this result. We detected 16 dispersers with a permissive p-value of 0.05 (Table 2). Among these, an even longer dispersal distance was detected. A putative disperser originated in an inland population (BAL) and moved to a coastal population (WEH), covering a distance of 188 km. While the latter method provides the same power as the L_home/L_max criterium with a p-value of 0.01, it also increases the error rate [39]. We further only consider migrants with p ≤ 0.01. Results of the Frequency based method are given in Additional file 1: S8.

**Table 2:** Identified long-distance dispersers at the sampling sites of *Hipparchia semele*

ID (Sex)	Sampling location	Putative origin	P-value	Dispersal distance (km)	Direction
WEH07 (m)	WEH	ZWI	0.028	61	C → C
SGD05 (f)	SGD	SLD	0.033	8	C → C
**TLM23 (f)**	**TLM**	**ZWI**	**0.006**	**142**	**C → I**
KAH14 (m)	KAH	ZWI	0.028	75	C → I
WEH05 (f)	WEH	BAL	0.029	188	I → C
**NIR12 (m)**	**NIR**	**TLM**	**0.000**	**26**	**I → I**
**KDM27 (f)**	**KDM**	**TLM**	**0.002**	**13**	**I → I**
**BBH07 (m)**	**BBH**	**KLB**	**0.005**	**32**	**I → I**
**SCB28 (f)**	**SCB**	**HHH**	**0.006**	**36**	**I → I**
**TLM01 (f)**	**TLM**	**MZP**	**0.006**	**22**	**I → I**
**ZBN08 (f)**	**ZBN**	**KAH**	**0.008**	**69**	**I → I**
WEV14 (f)	WEV	NIR	0.017	21	I → I
ZBN28 (m)	ZBN	TLM	0.017	6	I → I
KLB04 (m)	KLB	SCB	0.034	7	I → I
TEW14 (m)	TEW	NIR	0.047	39	I → I
BBH09 (m)	BBH	NIR	0.048	32	I → I

Results of assignment test using the Bayesian method in GENECLASS2. The individual identifier (ID) with its sex, sampling location, the most likely population of origin, the probability (p-value), the assumed dispersal distance in km and the direction of the dispersal event (C = coastal, I = inland) are given. Dispersal events with p-value ≤ 0.01 are indicated in bold

### Genetic diversity

We found higher levels of genetic diversity (in terms of allelic richness) in the inland populations than in the coastal populations (*Ar*: 3.26 and 2.83 resp., ANOVA test p < 0.05). Genetic diversity ranged from 2.8 to 4.3 alleles/locus and values of expected heterozygosity (*He*) ranged from 0.35 to 0.46. Corrected allelic richness values (*Ar*) ranged from 2.6 to 3.7 and the corrected number of private alleles (*Ap*) ranged from 0.0 to 0.1 (Table 1). Genetic diversity indices are shown in Additional file 1: S12. Evidence of inbreeding calculated with the R package DiveRsity was found in four of the five (80%) coastal populations (mean F_IS_: 0.161, range: 0.0859–0.277) and in twelve of the seventeen (70%) recent inland populations (mean F_IS_: 0.139, range: 0–0.259) (Table 1). The historical populations sampled in 2001 showed no evidence of inbreeding. After correcting F_IS_-values for the potential presence of null alleles, we still found evidence of inbreeding in two of the five (40%) coastal populations (mean corrected F_IS_: 0.086, range: 0.039–0.131) and in five of the seventeen (30%) recent inland populations (mean corrected F_IS_: 0.067, range: 0.016–0.137) (Additional file 1: S13). Only three coastal populations (SLD, TYZ and ZWI) showed weak evidence of genetic bottlenecks (under IAM p-values < 0.05, ZWI under TPM p-value < 0.05) (Additional file 1: S9). Estimation of *Ne* based on the sibship assignment method produced reliable results, as the two replicate runs yielded very similar values. *Ne* was estimated between 20 (ZWI) and 54 (ZBN) individuals using the Random Mating model (Table 1). We found a significant positive correlation between Ne and the area of a sample site (r = 0.64, p = 0.002). We further detected a positive trend between *Ar* and the area of a sample site (r = 0.40, p = 0.068) and a negative trend between Ar and the distance to the nearest population (r = − 0.40, p = 0.068). The other tested correlations were not significant.

### Population differentiation

The global genetic population differentiation was low (global G’_ST_ = 0.059 (95% C.I.: 0.046–0.073) and D_EST_ = 0.014 (95% C.I.: 0.009–0.020)). Regionally, genetic differentiation was even lower between the inland populations (regional G’_ST_ = 0.039 (95% C.I.: 0.025–0.054) and D_EST_ = 0.007 (95% C.I.: 0.002–0.013)) and slightly higher between the coastal populations (regional G’_ST_ = 0.060 (95% C.I.: 0.032–0.090) and D_EST_ = 0.017 (95% C.I.: 0.008–0.029)). Both global and regional differentiation were significant (G’_ST_: p < 0.001, global D_EST_: p < 0.001, regional D_EST_: p < 0.05). Overall, pairwise population G’_ST_-values ranged from − 0.012 to 0.160 (Additional file 1: S14). Regionally, pairwise population G’_ST_-values between inland populations ranged from − 0.012 to 0.114 and between coastal populations from 0.000 to 0.128. There was significant genetic differentiation (p < 0.05) between 97 of the 276 population pairs. IBD-analysis showed that genetic variability was only globally structured as indicated by the significant linear relationship (p = 0.00021, R^2^ = 0.28). Within the inland region, we found no significant isolation by distance (p = 0.185, R^2^ = 0.027). The IBD-analyses of the coastal region showed a positive trend (p = 0.087, R^2^ = 0.55). So there is weak evidence for isolation-by-distance in this region (Additional file 1: S15). The AMOVA indicated that the genetic variance was the highest within populations (95%), but only explained by region and population by 3% and 2% respectively (Additional file 1: S16). The Bayesian cluster analysis performed with BAPS (Additional file 1: S11) and STRUCTURE showed the highest probability for three clusters (K = 3) (Fig. 2). Individuals of the coastal populations were clearly clustered together. Since the inland populations showed more genetic admixture, STRUCTURE analyses showed no clear differentiation between the two inland clusters. Remarkably, the inland MEH population sampled in 2001 was assigned in the same cluster as the coastal populations, while the samples collected in MEH in 2020 clustered with neighbouring inland locations. Similarly, in the PCA populations were clustered according to region, separated by the first axis, with exception of the samples of the inland population MEH collected in 2001; the latter clustered together with the coastal populations (Additional file 1: S17).

## Discussion

We estimated long-distance dispersal based on microsatellite markers of the grayling *Hipparchia semele* inhabiting highly fragmented heathland or dune vegetation patches imbedded in an anthropogenic landscape in Belgium. Remarkably, maximum dispersal distance exceeded 100 km. Nevertheless, observed frequencies of long-distance dispersal events were low. The low genetic diversity reflected the recent population decline of the species in northern Belgium. While we found no strong evidence of severe bottlenecks, we detected inbreeding in several populations and we found low estimates for effective population sizes. Our results suggest that former studies likely have underestimated the dispersal distances that the grayling can cover. However, occasional long-distance dispersal events may be insufficient to preserve populations in this highly fragmented landscape in the long-term.

### High dispersal ability

The grayling is thought to be a fairly mobile butterfly species. Previous mark-release-recapture (MRR) studies observed covered distances of 1.2 km [55], 1.5 km [15] and 4 km [31] and colonisation distances up to 10–15 km [56]. We found seven putative first-generation dispersers (7 out of 599; 1.2%), of which six migrated between the inland heathland populations and one between a coastal and an inland population. The dispersal distances between the inland populations ranged from 13 to 69 km with a mean of 33 km, while the distance covered between the coastal population and the inland population was 142 km.

The dispersal distances observed in this study are substantially longer than the ones reported in previous MRR studies. In this study, we covered the entire distribution area of the grayling in northern Belgium (covering 12 625 km^2^). In contrast, previous MRR studies in the region [15, 31, 57] focussed on dispersal at a smaller spatial scale within one Belgian nature reserve. While the results of MRR studies can give more insight in local dispersal patterns (meters to several kilometres), the results based on genetic markers provide more insight in the dispersal ability of species at the regional spatial (landscape) scale (up to several hundreds of kilometres). Other studies found similar differences between dispersal distances obtained by observational methods such as MRR and those obtained from genetic marker studies (e.g. [58]). For example, for the sedentary butterfly *Phengaris (Maculinea) alcon*, Vanden Broeck et al. [21] found evidence of a dispersal distance of 2.9 km. In the same region, however, the maximum recorded movement of *P. alcon* from MRR studies was 0.5 km and the maximum distance recorded from spontaneous colonization data so far was 1.7 km [17]. To get a complete overview of the dispersal behaviour of a species, both direct (Mark-Release-Recapture) and indirect methods (genetic analyses) should ideally be implemented.

### Rare long-distance dispersal events

Although we provide evidence for dispersal ability of the grayling over unexpected long distances, the observed frequencies of long-distance dispersal events were low (1.2%). This observed low frequency of long-distance dispersal can, at least partly, be explained by the low genetic differentiation between populations, which limits the power of the assignment tests to detect dispersal events [39]. Furthermore, since we possibly did not sample all populations from which migrants may have originated (nearby populations in neighbouring countries (Fig. 1) and recently colonised populations within the region), we tested for the likelihood that an individual grayling was foreign-born, i.e. born in another population than where it was sampled [39]. The latter also results in a lower power to detect migrants compared to a sample including all potential source populations. Therefore, the real number of dispersers is likely to be underestimated in this study.

However, the majority of the detected long-distance dispersal occurred between inland populations, while no dispersal between the coastal populations was detected. Even with less strict detection methods, the vast majority of detected dispersal events occurred between the inland populations, with sparse dispersal events within the coastal region and between regions (Table 2; Additional file 1: S8). In contrast to the inland region, we found weak evidence of isolation-by-distance in the coastal region, supporting the observed lack of dispersal events between sampled locations within the coastal region. Increasing long-distance dispersal events would decrease the isolation and would likely lower the strength of inbreeding among the coastal populations. For the inland region, the sampled locations consist of a metapopulation with interaction by gene flow between the subpopulations. Here, sampled locations are less isolated compared to the sampled locations of the coastal region, despite longer distances between inland populations. This indicates a higher connectivity and lower extinction risk for the grayling in the inland region compared to the coastal region. The differences in dispersal events observed between the coastal and inland region could be explained by differences in the landscape matrix (for detailed maps, see Additional file 1: S18). Low long-distance dispersal events in the Belgian coastal region can likely be attributed to the high levels of urbanization. Buildings, parking lots and roads separate small patches of grey dune habitat by many kilometres [59] and may act as barriers limiting gene flow. A study on the effect of urbanization on the species richness of different taxa found that butterflies, and the most mobile and specialist species in particular, were strongly negatively affected by urbanization [60]. However, our findings are in contrast with the results of a population genetic study of the endemic butterfly *Atrytonopsis* sp. on the highly urbanized coast of North Carolina (USA). Based on Amplified Fragment Length Polymorphic (AFLP) molecular markers, Leidner and Haddad [61] found that not coastal urbanization but natural barriers (ocean and forests) limited butterfly dispersal between coastal populations. It must be noticed that, in the latter study, conclusions on actual migration rates were based on the population genetic structure obtained by the program STRUCTURE [53] and on Isolation-by-distance (IBD) analyses. These methods are however considered unsuitable for making reliable deductions about actual dispersal events. Generally, STRUCTURE yields poor individual assignments to source populations and it does not reliably recover the actual population structure when sampling is unbalanced [62]. Furthermore, patterns of IBD can arise from limited dispersal but also from historical demographical processes such as founder effects and historical gene flow rather than contemporary dispersal rates [52, 63]. Due to these concerns, we used genetic assignment tests instead. Genetic assignment tests are based on the distribution of observed allele frequencies to draw inference about where individuals were or were not born, allowing direct, real-time estimates of dispersal [39]. These assignment tests are useful for the identification of immigrant individuals in the current generation and thus the potential for contemporary gene flow, which was the interest of this study.

In contrast to the coastal region, the grayling inland populations are principally surrounded by a matrix of woodland, heathland and meadows. Woodlands are often considered to be dispersal barriers for butterflies of open habitats (e.g. heathlands, grasslands), but Nowicki et al. [64] showed that long-distance butterfly dispersal is not always prevented by forests. Moreover, towards the southern and eastern part of the grayling’s distribution range, woodland and trees are a part of its habitat [65]. The inland populations may therefore be located in a more favourable matrix compared to the coastal populations. We can however only speculate about possible barriers in the different regions. A study on landscape genetic analysis, which takes spatial structures into account, should provide more robust evidence for potential barriers for the grayling.

### Fragmentation effects on genetic diversity

When habitat fragmentation hampers gene flow, populations can suffer from loss of genetic diversity reflected by low effective population sizes, resulting in inbreeding and extinction risks. We did find evidence of inbreeding in several populations and low estimates for effective population sizes (range: 20–54 individuals). Inbreeding increases levels of homozygosity and exposes deleterious recessive alleles, which can result in inbreeding depression and reduced population fitness [5, 66, 67]. These negative consequences on the population level can have implications for metapopulation dynamics [68]. As a loss of populations reduces the total number of migrants, increased population extinctions caused by inbreeding would decrease the colonization probability of unoccupied habitat patches [68]. An entire metapopulation, and especially a small metapopulation, might thus suffer from an increased extinction risk caused by inbreeding effects [68, 69].

For further investigating the potential effects of fragmentation on genetic diversity, we included samples collected two decades ago (dated 2001) from two inland populations (MEH and TEB) and compared the genetic diversity with recent samples of the corresponding locations (dated 2020). Levels of genetic diversity, in terms of effective population size and allelic richness, were similar between the recent and historical samples for both locations. However, for the location MEH, there was no evidence of inbreeding in the historical sample, while we found weak evidence of inbreeding in the current population. This may indicate that the levels of diversity did not change over the past two decades, but that a consistent low average population size (we estimated low effective population sizes and the census population sizes have been decreasing over decades [30]) caused an increase in inbreeding over time. Additionally, a remarkable result is the clustering of the 2001 population of MEH with the coastal populations. This might indicate that the two regions were more genetically similar in the past likely because of a higher connectivity between the regions.

Previous studies on the two heathland butterfly species *Phengaris alcon* [21] and *Plebejus argus* (unpublished data) occured in the same inland Campine region of this study. The results of these studies and our study on *H. semele* were obtained using the same methods. *P. alcon* is listed as ‘Critically endangered*’*, while *P. argus* is listed as ‘Endangered’ in Flanders [30]. In terms of effective population size and genetic diversity, we found similar results to both previously studied species. The inbreeding coefficients in our study were similar to the ones found in the *P. argus* populations. The study on *P. argus* also found a low number of dispersers within the inland region and no significant Isolation-by-distance.

Our inbreeding values should however be interpreted with caution. While we took into account the presence of null alleles during the selection of the loci for analyses, there is still evidence for null alleles in a small percentage of loci *x* population combinations. The presence of null alleles causes an increase in homozygosity, which means that levels of inbreeding could be inflated (e.g. [44]). Additionally, substructure within populations can also lead to a deficiency of observed heterozygotes (the Wahlund effect [70]), and an increased coefficient of inbreeding [38, 67]. Though we cannot exclude a reduction in heterozygosity caused by subpopulation structure in some of the sampled locations, individuals were generally caught in fairly small sampling areas (Additional file 1: S2). *H. semele* is also a mobile species that can easily move distances of 100 m and more within a couple of days [15], covering a large area within a sampling location.

### Implications for conservation

Our study corroborates the results of demographic studies that show strong population declines of the grayling principally caused by habitat loss and fragmentation, particularly in North-West Europe (e.g. [6, 7, 25, 30, 71]). Climate change may also have a negative impact on reproductive success [72], as extreme droughts have already occurred in Belgium during the last few years (e.g. 2018 and 2019). Conservation actions are needed to prevent a further decline and local population extinctions of the grayling in northern Belgium.

Based on the habitat characteristics and the genetic differentiation and mobility, we can assign grayling populations in northern Belgium to two different Functional Conservation Units (FCUs) (cf. [17]); the coastal conservation unit (FCU 1) and the inland heathland conservation unit (FCU 2). Although these conservation units, separated by at least 70 km, are potentially connected by gene flow and thus not completely isolated from each other, we consider them as different spatial entities in which specific management and restoration measures should be implemented. Estimates of the effective population sizes were higher in larger sample locations. Additionally, the allelic richness of the populations showed a trend of being higher in larger areas and in populations with shorter nearest neighbour distances. Within each conservation unit, management actions should therefore focus on enlarging actual and restore potential habitat areas.

For the coastal conservation unit (FCU 1), as current inter-population connection is likely extremely low, we suggest that the emphasis should be on creating new habitat between existing populations in order to improve population network connectedness. Furthermore, translocation actions such as reinforcements should be considered, as spontaneous exchange of individuals between populations is likely hindered by anthropogenic barriers to gene flow along the Belgian coast and because of the observed inbreeding. The inland conservation unit (FCU 2) consists of a larger metapopulation, in terms of the number of demes and occupied territory, and connectivity between sub-populations is likely higher and less hindered by anthropogenic barriers compared to the coastal populations. However, also for FCU 2, restored and/or new heathland habitat have likely a low chance to be colonized spontaneously within a short time frame. Over the past years, there has been a strong decline of the grayling in Northern Belgium [30], which is most noticeable in the central and western part of the inland region. Relying on spontaneous colonisation and gene flow for the recovery of populations would therefore be too a considerable risk. Therefore, we recognize targeted re-introductions and translocations as valuable management actions to improve the connection between the populations in the western and eastern part of the inland region.

Translocations should be conducted according to IUCN guidelines [73]. To avoid a high risk of outbreeding depression [74], founder populations should consist of individuals from the same conservation unit. Founder individuals should be chosen from non-inbred populations with high genetic diversity, as inbreeding and reductions in genetic diversity are likely to lower fitness and thus the persistence of the founder population [5]. For the coastal conservation unit (FCU 1), Schipgatduinen (SGD) could act as a source sub-population for translocations as it is one of the largest suitable areas (the risk of damaging the local populations is low) and it is a population with a low inbreeding value. However, levels of genetic diversity in SGD are low and therefore combining different sub-populations as a source could be considered as an alternative. For the inland conservation unit (FCU 2), the sub-population Zwarte Beek Noord (ZBN) appears the best source population for translocations. It has the highest estimated level of genetic diversity and the highest estimated effective population size of all the sub-populations. To assess translocation success, an appropriate monitoring scheme needs to be implemented.

## Conclusions

This genetic study on the grayling showed that dispersal distances covered by grayling butterflies were much larger than previously assumed. Long-distance movements are however scarce. Estimated effective population sizes were low and several populations showed evidence for inbreeding, which reflect the consequences of strong population declines during the last decades. Although genetic differentiation was found to be low, both within as between regions, there was a significant differentiation between the coastal and inland region. Therefore, we suggest to treat the two regions as as two separate conservation units. Urgent measures (nature management, translocations, reinforcement or supplementation) are needed for the sustainable conservation of the species in northern Belgium.

## Supplementary Information

**Additional file 1.** **S1.** Distribution of *Hipparchia semele* in North-West Europe. **S2. **Samples and sampling sites of *Hipparchia semele*. **S3.** DNA extraction. **S4.** Microsatellite development. **S5.** Microsatellite loci and PCR-conditions. **S6.** Estimation of the frequency of null alleles with the Dempster method. **S7. **Hardy-Weinberg equilibrium and Linkage Disequilibrium testing. **S8.** Population assignment test: Frequency based method. **S9.** Genetic bottlenecks. **S10.** Estimation of effective population size (Ne). **S11. **STRUCTURE and BAPS analysis. **S12.** Genetic diversity statistics. **S13.** The effect of null alleles on the FIS-values. **S14.** Pairwise G’ST values. **S15.** Isolation-by-distance analyses. **S16.** Levels of hierarchical structuring within populations, among populations and among regions estimated by analyses of molecular variance (AMOVA). **S17.** Plots of the Principal Component Analysis (PCoA). **S18.** Detailed maps of the land-use in northern Belgium.

## Data Availability

The dataset supporting the conclusions of this article is available in the Dryad repository, https://doi.org/10.5061/dryad.5tb2rbp4h.

## Abbreviations

AMOVA: Analysis of molecular variance
FCU: Functional conservation unit
IBD: Isolation-by-distance
LD: Linkage disequilibrium
MRR studies: Mark-release-recapture studies
PCA: Principal components analysis
