# Supplementary material for: Occasional long-distance dispersal may not prevent inbreeding in a threatened butterfly
Source: BMC Ecol Evol. 2021 Dec 27;21:224. doi: 10.1186/s12862-021-01953-z (PMC8711176; doi:10.1186/s12862-021-01953-z)
Supplement: Supplementary file 1 — Additional file 1. S1. Distribution of Hipparchia semele in North-West Europe. S2. Samples and sampling sites of Hipparchia semele. S3. DNA extraction. S4. Microsatellite development. S5. Microsatellite loci and PCR-conditions. S6. Estimation of the frequency of null alleles with the Dempster method. S7. Hardy-Weinberg equilibrium and Linkage Disequilibrium testing. S8. Population assignment test: Frequency based method. S9. Genetic bottlenecks. S10. Estimation of effective population size (Ne). S11. STRUCTURE and BAPS analysis. S12. Genetic diversity statistics. S13. The effect of null alleles on the FIS-values. S14. Pairwise G’ST values. S15. Isolation-by-distance analyses. S16. Levels of hierarchical structuring within populations, among populations and among regions estimated by analyses of molecular variance (AMOVA). S17. Plots of the Principal Component Analysis (PCoA). S18. Detailed maps of the land-use in northern Belgium. [file 12862_2021_1953_MOESM1_ESM.pdf]

Additional file 1

Occasional long-distance dispersal may not prevent inbreeding in a threatened butterfly

Annelore De Ro[1], An Vanden Broeck[1], Leen Verschaeve[1], Ilf Jacobs[2], Filiep T'Jollyn[3], Hans Van Dyck[4] & Dirk Maes[3]

1 Research Institute for Nature and Forest (INBO), Gaverstraat 4, B-9500 Geraardsbergen, Belgium; annelore.dero@inbo.be; an.vandenbroeck@inbo.be; leen.verschaeve@inbo.be

2 Natuurpunt Studie, Coxiestraat 11, B-2800 Mechelen, Belgium; ilf.jacobs@natuurpunt.be

3 Research Institute for Nature and Forest (INBO), Herman Teirlinckgebouw, Havenlaan 88 box 73, B-1000 Brussels, Belgium; filiep.tjollyn@inbo.be; dirk.maes@inbo.be

4 Behavioural Ecology and Conservation Group, Biodiversity Research Centre, Earth and Life Institute, Université Catholique de Louvain (UCLouvain), Croix du Sud 4-5, B-1348 Louvain-la-Neuve, Belgium; hans.vandyck@uclouvain.be

Additional file

S1. Distribution of Hipparchia semele in North-West Europe 3
S2. Samples and sampling sites of *Hipparchia semele* 4
S3. DNA extraction 6
S4. Microsatellite development 7
S5. Microsatellite loci and PCR-conditions 8
S6. Estimation of the frequency of null alleles with the Dempster method 9
S7. Hardy-Weinberg equilibrium and Linkage Disequilibrium testing 10
S8. Population assignment test: Frequency based method 13
S9. Genetic bottlenecks 14
S10. Estimation of effective population size (*Ne*) 15
S11. STRUCTURE and BAPS analysis 17
S12. Genetic diversity statistics 20
S13. The effect of null alleles on the F_{IS}-values 21
S14. Pairwise G'_{ST} values 24
S15. Isolation-by-distance analyses 25
S16. Levels of hierarchical structuring within populations, among populations and among regions estimated by analyses of molecular variance (AMOVA) 27
S17. Plots of the Principal Component Analysis (PCoA) 28
S18. Detailed maps of the land-use in northern Belgium 29
References 31

S1. Distribution of *Hipparchia semele* in North-West Europe

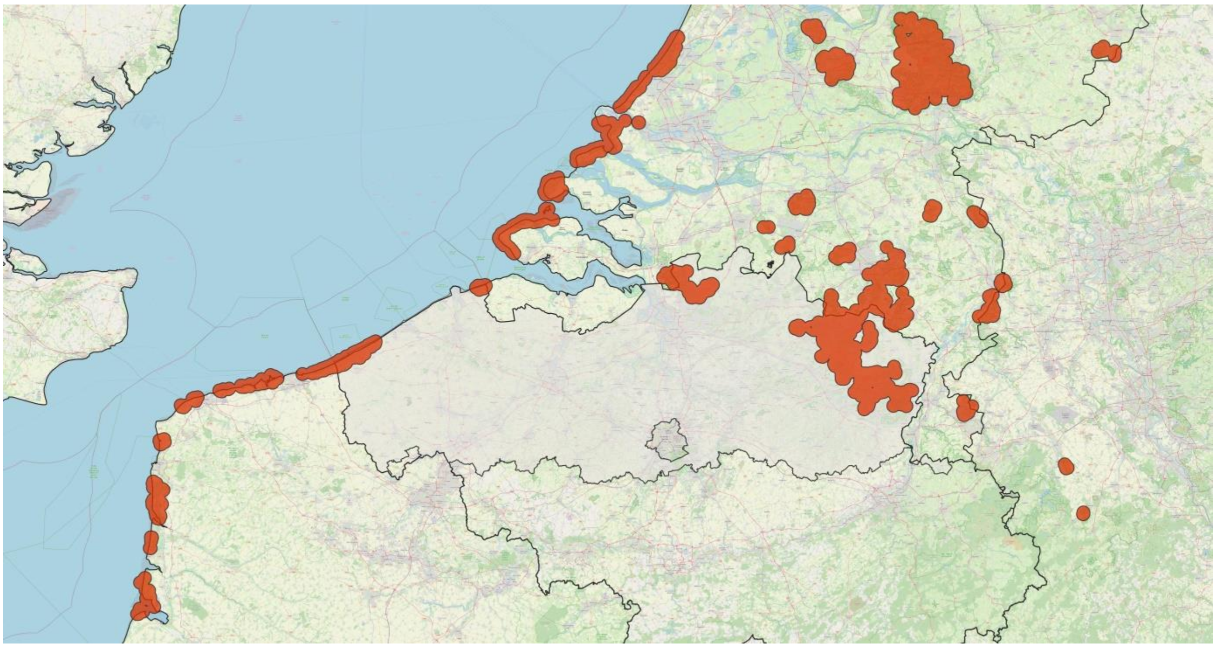

Figure S1 Map of North-West Europe with clustered observations of the grayling since 2011 (red polygons) with a 2.5 km radius.

The distribution data used for this map was downloaded from GBIF (https://www.gbif.org/), iNaturalist (https://www.inaturalist.org/) and observado (https://observation.org/).

S2. Samples and sampling sites of *Hipparchia semele*

Table S1 Sample sites, site area, the area where sampling took place, the maximum distance between samples within a site, the distance to the nearest population, the number of *Hipparchia semele* individuals sampled and number of samples successfully analysed.

Location	Location code	Region	Sampling date	Coordinates	Area Site (m²)	Area Sampled (m²)	Max. distance between samples within a site (m)	Distance to nearest population (m)	# Samples	# Samples analysed
**2001**										
1. Mechelse Heide	MEH	Inland	-	-	3 791 324	-	-	-	24	24
2. Teutelberg	TEB	Inland	-	-	119 902	-	-	-	20	18
**Total 2001**		2							44	42
**2020**										
3. Westhoek	WEH	Coast	29/07/2020	51.108637N/2.569824E	2 049 699	116 817	430	6 132	30	30
4. Schipgatduinen	SGD	Coast	30/07/2020	51.124790N/2.645055E	348 106	4197	89	1 704	30	29
5. Ter Yde – Zeebermduinen	TYZ	Coast	30/07/2020	51.136799N/2.700728E	774 257	13 342	702	1 704	30	30
6. Sint-Laureinsduinen	SLD	Coast	29/07/2020	51.161076N/2.751019E	463 068	14 604	246	3 455	30	30
7. Zwin	ZWI	Coast	27/07/2020	51.364605N/3.349195E	524 685	10 296	200	45 049	30	30
8. Kalmthoutse Heide	KAH	Inland	06/08/2020	51.401485N/4.435003E	7 434 512	205 215	894	46 730	30	29
9. Klein Schietveld	KLS	Inland	-	-	-	-	-	-	0	0
10. Groot Schietveld	GRS	Inland	24 & 25/08/2020	-	-	-	-	-	0	0
11. Niras	NIR	Inland	23/07/2020	51.224171N/5.073072E	290 086	19 070	526	5 218	30	26
12. Keiheuvel – De Most	KDM	Inland	21 & 23/07/2020 & 05/08/2020	51.182834N/5.220805E	1 070 387	124 152	958	1 292	30	26
13. Balimgronden	BAL	Inland	20/07/2020	51.197944N/5.263132E	4 258 107	19 118	210	1 292	30	29
14. Maatheide	MAH	Inland	05/08/2020	51.228844N/5.257022E	-	-	-	-	2	0
15. Hageven	HAG	Inland	13 &	51.269123N/5.426589E	1 021 629	15 549	1193	5 642	30	28

			16/08/2020							
16. Beverbeekse Heide	BBH	Inland	13 - 16/08/2020	51.293304N/5.512010E	522 858	487 435	1914	5 642	30	27
17. Weyersvlakte	WEV	Inland	10/08/2020	51.13657N/5.337216E	3 991 023	27 215	316	1 138	30	30
18. Zwarte Beek – Noord	ZBN	Inland	10/08/2020	51.108760N/5.306172E	15 345 420	26 421	350	667	30	27
19. Zwarte Beek – Zuid	ZBZ	Inland	10/08/2020	51.091782N/5.346917E	5 578 403	35 197	461	667	30	25
20. Terril Lindeman	TLM	Inland	04/08/2020	51.056843N/5.326924E	191 168	20 069	283	1 847	30	28
21. Militair Schietveld – Houthalen-Helchteren	HHH	Inland	19 & 24/08/2020	51.045418N/5.456590E	12 381 050	73 117	542	4 319	30	28
22. Terril Winterslag	TEW	Inland	21 & 22/07/2020	50.984821N/5.477813E	743 211	53 548	315	644	30	24
23. Schemmersberg	SCB	Inland	12/08/2020	50.980525N/5.457737E	170 879	31 021	336	644	30	29
24. Klaverberg	KLB	Inland	22/07/2020	51.009792N/5.548819E	972 762	36 705	802	4 319	30	23
25. Mechelse Heide	MEH	Inland	12/08/2020	50.976566N/5.650973E	3 791 324	247 521	3132	3 133	30	20
26. Teutelberg	TEB	Inland	30/07/2020 & 04/08/2020	51.003086N/5.686929E	119 902	103 988	673	3 133	25	21
27. Molse Zandputten	MZP	Inland	05/08/2020	51.224333N/5.153156E	72 339	6434	127	5 218	30	30
**Total 2020**		24							657	599
**Total**		26							701	641

S3. DNA extraction

Before extraction of genomic DNA, each wing fragment was air-dried for 1 hour to be sure that all the 96% ethanol was vaporised. Then, each fragment was first homogenized in 5 µL proteinase K (>600mAU, Qiagen) for a couple of minutes to let the product take effect. Next, 100 µL 6% Chelex InstaGene Matrix solution (Biorad) was added. The samples were incubated at 56 °C for 60 min. while being stirred at 750 rpm, boiled at 99 °C for 15 min. while being stirred at 400 rpm, and then cooled down for a few minutes and centrifuged at 14000 rpm for 3 min. The supernatant was stored at -20 °C.

S4. Microsatellite development

Microsatellite development and genotyping services were carried out by AllGenetics & Biology SL (www.allgenetics.eu). Briefly, a genomic DNA library was prepared using the extracted DNA as input for the Nextera XT DNA Library Preparation kit (Illumina), which was used strictly following the manufacturer's instructions.

The library was enriched with the following microsatellite motifs: AC, AG, ACG, and ATCT. The library was then sequenced in the Illumina MiSeq platform (PE300). The library produced 5,191,783 paired-end reads. The quality of the raw sequencing data was checked using FastQC 0.11.15. Reads that showed low-quality were identified and trimmed using Trimmomatic 0.36 (1, Bioinformatics btu170). Then, a second quality check was performed using FastQC 0.11.15 to make sure only high-quality reads were used for the next steps. Reads were processed in Geneious 8.1.9 (Biomatters Ltd) and using in-house developed scripts. Primer design was carried out in Primer 3 (2, 3) implemented in Geneious 10.2.3. These primer pairs hybridise at the flanking regions of microsatellite motifs.

A total number of 73 primer pairs organised in 22 multiplexes were biologically tested and checked for polymorphism. PCRs were carried out following Schuelke (4). The oligonucleotide tails used were the universal sequences M13 (5' GGA AAC AGC TAT GAC CAT 3'), CAG (5' CAG TCG GGC GTC ATC 3'), and T3 (5' AAT TAA CCC TCA CTA AAG GG 3'). The three oligonucleotides were labelled with the HEX dye, the FAM dye, and the TAMRA dye, respectively. PCRs were performed in a final reaction volume of 12.5 µL, containing 1 µL of DNA (10 ng/µL), 6.25 µL of the Type-it Microsatellite PCR Kit (Qiagen), 4 µL of PCR-grade water, and 1.25 µL of the primer mix. The optimal PCR protocol consisted in an initial denaturation step at 95 °C for 5 min, followed by 30 cycles of 95 °C for 30 s, 57 °C for 90 s, 72 °C for 30 s; 8 cycles of 95 °C for 30 s, 53 °C for 90 s, 72 °C for 30 s; and a final extension step at 68 °C for 30 min. PCR products were subsequently subjected to fragment analysis on an ABI 3730xl DNA Analyzer (Applied Biosystems, USA). Alleles were called using Geneious 10.2.3.

Out of the 73 tested microsatellite loci, a total number of 18 primer pairs amplified polymorphic microsatellite loci. These 18 primer pairs were re-organised into 6 new multiplexes. New PCRs were carried out following the protocol described above.

S5. Microsatellite loci and PCR-conditions

Table S2 Polymorphic nuclear microsatellite loci developed for *Hipparchia semele* and used in this study.

	Locus	Sequence motif	Product size	Primer	Ta (C°)	Dye	Multiplex
**1**	AG_Hse_007	AG	57 - 97	F: AGCACGTAGAGCTGTCGGTT	64.2	FAM	2
				R: CAGGTGCCTTCACTCTCACA			
**2**	AG_Hse_039	AC	80 - 120	F: GTAACTAGCCACGGCCAAAG	61.5	FAM	3
				R: GGTTCATTCATTGTTGCGTG			
**3**	AG_Hse_072	AAG	110 - 140	F: CTGTGGAAGTCCATGCAAGA	61.7	VIC	3
				R: CGCGTGGAATCTGACTGATA			
**4**	AG_Hse_076	AC	140 - 160	F: GGTACATTTGTTGTGGCGTG	63.3	NED	2
				R: CTGACAGGATAGCGACCGAT			
**5**	AG_Hse_185	AC	100 - 120	F: AGCAAGCTCTCGCTCACACT	61.2	VIC	2
				R: CGAAACATTGCTAGTTGCCA			
**6**	AG_Hse_186	AC	204 - 299	F: TTTGGCGGTTTGGAATAGAG	60.5	VIC	1
				R: ATGGTGAAGTGGACAACGTG			
**7**	AG_Hse_210	CCG	160 - 211	F: GAAGCGAGCCACTACGAGTC	63.3	NED	3
				R: TGTGCTCGAACCCTTGGTAT			
**8**	AG_Hse_214	ACC	91 - 142	F: GTCAGAGGGTCAGGAGTTCG	61.0	NED	3
				R: GCCAGAGCGGTTTAAAGAAA			
**9**	AG_Hse_229	ACGAT	133 - 148	F: GGAGACAGTTGGACAGGTGG	64.3	PET	2
				R: CACATGTGGGCCTATGTCAC			
**10**	AG_Hse_269	AT	134 - 156	F: CTACGCACAAGTGGCATTTC	63.0	VIC	1
				R: GATCGCAAGGAAGTGTAGGC			
**11**	AG_Hse_343	ACC	127 - 136	F: TGACAGGGTATCGGTCTGAA	61.2	PET	1
				R: GCTTACCCAGGTGGCAGATA			
**12**	AG_Hse_350	ACGG	126 - 146	F: CTGTGAAATGCCGGTTACAA	60.9	NED	1
				R: GGACAATTGGTGTTTGACGA			
**13**	AG_Hse_369	AC	88 - 102	F: TTTCAAGACACAAGACGGCA	59.7	PET	3
				R: TCTCGATTAGTTGGATGCGA			
**14**	AG_Hse_371	AAAC	76 - 98	F: ATTCGAAAGGTTCCGTACCA	61.0	VIC	1
				R: GTCGATCCGTAGATCGGTTG			
**15**	AG_Hse_376	CG	236 - 254	F: TCCAGATGAAGTGGACGTGA	61.8	FAM	2
				R: ACTAGGACCGGTCGAGTGTC			
**16**	AG_Hse_421	ATCC	80 - 100	F: TTATTCCAGTCAGTCGACGC	61.9	NED	2
				R: CACGTCTTTGACGGTAGGGT			
**17**	AG_Hse_426	AC	214 - 261	F: ATTCGGCTGAAAGGAATGG	61.2	PET	3
				R: TATCGTCAGGTGCATCGTGT			
**18**	AG_Hse_454	CG	116 - 118	F: GCTGTACTACTCCAACGCGA	64.0	FAM	1
				R: ACAGAGGGACGCTGTAGTGG			
**19**	AG_Hse_471	AAG	155 - 205	F: GCGTTGTTCACCGAAATATG	60.9	FAM	1
				R: CAAACAAGTCGCAGGGAACT			
**20**	AG_Hse_489	ATC	142 - 175	F: GAATCGAGTGGATGCGAAA	60.1	PET	3
				R: GTGGCCTACACGGCTGTTAT			

PCR conditions

20 primer pairs with 5´ fluorescently labelled primers (dyes: FAM, VIC, NED, PET) were organised into 3 multiplexes using Multiplex Manager 1.2 (5). For each DNA sample, the polymorphic nuclear microsatellites were amplified using the QIAGEN multiplex Master mix (hotstar) and standard PCR conditions; initial denaturation for 15 min at 95 °C followed by 35 cycles of 30 s at 95 °C, 30 s at locus-specific annealing temperature and 30 s extension at 72 °C, finishing with elongation of 30 min at 72 °C using a Biometra Thermocycler. Total reaction volume was 10 µL of which 2 µl was template DNA. PCR products were run on an ABI 3500 analyser with the GeneScan-600 LIZ size standard and analysed using Geneious Prime 2019.3.2 (https://www.geneious.com).

S6. Estimation of the frequency of null alleles with the Dempster method

Table S3 Estimated frequency of null alleles calculated with the Dempster method in GENEPOP v4.3. Frequencies > 0,20 and loci used in further data analyses are indicated in bold. The Dempster method employs an iterative EM (expectation and maximization) approach to find the maximum likelihood estimate of null allele frequency (6).

	**Hse_454**	Hse_471	Hse_371	**Hse_269**	Hse_186	**Hse_350**	**Hse_343**	**Hse_007**	**Hse_376**	**Hse_229**	Hse_214	Hse_210	**Hse_369**	**Hse_489**	Hse_426
SGD	/	**0.3111**	0.1087	0.0000	**0.2209**	0.0000	**0.3412**	0.099	0.1125	0.0678	0.1572	0.1443	0.0000	0.0586	**0.3545**
SLD	/	**0.4565**	0.1054	0.0603	**0.2924**	0.0938	**0.2522**	/	0.0676	0.0000	**0.2602**	0.0886	0.0278	0.1528	**0.2993**
TYZ	/	0.1381	**0.2890**	0.0678	0.1814	0.0252	**0.3328**	0.0684	/	0.0634	**0.2435**	**0.2644**	0.0000	0.1325	0.1934
WEH	/	**0.2889**	**0.3505**	0.1871	**0.3225**	0.0345	**0.2875**	0.1329	0.0000	0.0000	**0.2037**	0.0334	0.0000	0.1083	**0.3778**
ZWI	/	**0.3123**	**0.2863**	0.0611	0.1877	0.0255	**0.4704**	0.0563	0.0250	0.0095	0.1440	0.1506	0.0000	**0.2119**	**0.2296**
BAL	/	**0.2576**	0.1168	0.0683	**0.2790**	0.0675	**0.3163**	0.1302	0.1077	0.0749	0.1745	0.1866	0.0000	0.1669	**0.3632**
BBH	0.1925	0.1129	**0.2535**	0.0255	**0.2623**	0.0000	0.1048	0.0000	0.1213	0.0696	**0.3252**	**0.2502**	0.0000	0.1875	**0.2712**
HAG	/	0.1914	**0.2276**	0.0569	**0.2812**	0.0457	**0.2235**	0.1568	0.1429	0.0185	**0.3404**	**0.2747**	0.0000	0.0917	**0.2790**
HHH	/	**0.3041**	**0.2147**	0.0000	**0.2740**	0.0588	0.0149	0.103	0.0000	0.0891	**0.3373**	**0.2703**	0.0000	0.0676	0.1362
KAH	/	**0.2711**	**0.2581**	0.0313	**0.3767**	0.0000	**0.2020**	0.1065	/	0.1625	0.1107	0.0837	0.0700	0.1560	**0.2853**
KDM	/	**0.2222**	**0.2892**	0.0000	**0.4069**	0.0298	0.1756	0.1011	/	0.0828	**0.2931**	0.1141	0.0000	0.1641	**0.3113**
KLB	0.2085	**0.2222**	**0.4825**	0.0000	**0.3233**	0.0000	0.1670	0.1833	/	0.1030	0.1529	**0.2306**	0.0000	**0.2828**	**0.2778**
MEH	/	**0.4379**	**0.2500**	0.0809	**0.3103**	0.0000	0.1484	**0.2174**	/	0.0000	**0.3680**	**0.2060**	0.056	**0.2260**	**0.3350**
MEH2001	/	**0.2744**	0.1117	0.0000	**0.2483**	0.0483	**0.2958**	0.0000	0.0846	0.0332	0.1008	0.1770	0.0116	0.0602	0.1809
MZP	0.0000	**0.2741**	**0.2722**	0.0668	0.1721	0.0000	0.1271	0.0000	**0.2553**	0.0000	**0.2124**	**0.2177**	0.0000	**0.2371**	**0.3983**
NIR	**0.2722**	**0.3104**	0.0531	0.0000	**0.2393**	0.0312	**0.2295**	0.1011	0.1493	0.1030	**0.2002**	**0.3499**	0.0000	**0.2124**	**0.3445**
SCB	0.0000	**0.2750**	**0.3306**	0.0540	**0.2959**	0.0000	0.0000	0.0000	/	0.0672	0.1260	0.1841	0.0000	0.1462	**0.5891**
TEB	0.0000	**0.2727**	**0.2007**	0.0469	**0.3196**	0.0000	0.1205	0.0000	/	**0.2325**	**0.2954**	0.1978	0.0044	0.0675	**0.2801**
TEB2001	0.0000	0.0743	**0.2382**	0.0596	**0.2819**	0.0000	0.1978	0.0000	0.0000	0.0000	0.1400	**0.2020**	0.000	0.1550	**0.3720**
TEW	0.0000	**0.2259**	**0.2215**	0.0000	**0.2950**	0.0000	0.1420	**0.2413**	0.0000	0.0581	0.0900	0.1280	0.0000	0.1178	**0.4877**
TLM	**0.2673**	**0.3050**	0.1649	0.0725	**0.2284**	0.0000	0.1645	**0.2782**	0.1274	0.0146	**0.2334**	**0.2262**	0.0000	0.1474	**0.2614**
WEV	0.1826	**0.2516**	**0.2089**	0.0439	**0.3022**	0.1197	0.0371	0.1231	/	0.0711	**0.3052**	0.1566	0.0000	0.0702	**0.3590**
ZBN	0.1890	**0.2591**	**0.2540**	0.0000	**0.2982**	0.0476	0.0233	0.0000	0.1346	0.0593	**0.2572**	0.1767	0.0000	0.0667	**0.2918**
ZBZ	/	**0.3290**	**0.3003**	0.0000	0.1527	0.0000	0.0000	0.0000	**0.2037**	0.0428	**0.3101**	**0.2513**	0.0000	0.1261	**0.2431**

S7. Hardy-Weinberg equilibrium and Linkage Disequilibrium testing

In total, 701 butterfly wing clips were genotyped. Five microsatellite loci (*Hse_039, Hse_072, Hse_076, Hse_185* and *Hse_421*) were discarded from the analyses due to a difficulty in scoring, resulting in a total of 15 microsatellite loci included in the study. Based on the replicated samples, the mean genotyping error was 1.6%. Removing 48 (6.8%) samples with missing data at more than 3 loci resulted in 653 unique genotypes. These removed samples included the only two samples of the population Maatheide (code: MAH), resulting in a total of 24 sampling locations used in the following analyses. Next, we examined the assumptions of Hardy-Weinberg equilibrium at individual loci and of no linkage disequilibrium (LD) at pairs of loci because these were fundamental assumptions of the following genetic analyses.

For 24 populations, we tested 360 locus x population combinations for a significant departure from Hardy-Weinberg proportions (HWP). After sequential Bonferroni correction (Bonferroni adjusted $p < 0.0001$), 109 (30%) combinations departed significantly. The next eight loci showed significant departure from HWP: *Hse_186, Hse_426, Hse_471, Hse_214, Hse_210, Hse_371, Hse_489* and *Hse_343*; for the former two we found significant departures in almost every population. Next, we calculated moderate to high estimates for null allele frequencies ($r > 0.20$) for 36% of the locus x population combinations which may have caused the observed departures from HWP. Significant linkage disequilibrium (LD) ($p < 0.0001$) was only observed between *Hse_471* and *Hse_210* in the population WEV.

To check the influence of the loci with deviation from HWP and high frequencies of null-alleles on further analyses, we followed recommended steps by Waples (7). Robustness of the results was evaluated by comparing results for overall (G'_{ST}, D_{EST}) and population-specific genetic diversity (F_{IS}, *Ho, He, Ar*), with and without each of these specific loci (Table S4, Table S5). Loci were deleted from the dataset in an order so that the loci that showed the most evidence for departure from HWE and for null-alleles were deleted first. For further analyses to be reliable, we excluded the next six loci: *Hse_186, Hse_426, Hse_471, Hse_214, Hse_210 and Hse_371*. We chose the dataset with 9 SSR loci, as previous datasets still contained to much loci with HW-departures and null-alleles for the reliability of the analyses. G'st values were not robust after discarding *Hse_489* (dataset with 8 SSR).

After removing these loci, only three locus x population combinations (1%) departed significantly from HWP. Only 7% of the combinations showed high estimates for null allele frequencies ($r > 0.20$) and no population showed high estimates for more than three loci.

Table S4 Compilation of the mean diversity indices and inbreeding coefficients obtained for the different datasets. The data column indicates how many loci were still present in the dataset and which locus was excluded relative to the bigger dataset. With: the mean number of individuals genotyped per locus (*N*); the mean number of alleles per locus and corrected for sample size (based on a minimum of 13 individuals) (*Ar*); the observed heterozygosity (*Ho); the expected heterozygosity (*He*); the mean Wright's inbreeding coefficient per locus (F_{IS}); the lower limit of the 95% Confidence Interval of the corresponding F_{IS}-value (F_{IS}_*Low*);); the upper limit of the 95% Confidence Interval of the corresponding F_{IS}-value (F_{IS}_*High*); and the number of populations for which evidence of inbreeding was found.

Data	N	Ar	Ho	He	F_{IS}	F_{IS}_Low	F_{IS}_High	Evidence for inbreeding
Total SSR data (15 loci)	24.84	2.75	0.34	0.54	0.37	0.28	0.45	24
14 SSR (excl. *Hse_186)*	24.92	2.66	0.34	0.52	0.34	0.25	0.43	24
14 SSR (excl. *Hse_426*)	25.09	2.67	0.34	0.52	0.34	0.24	0.42	24
14 SSR (excl. *Hse_471*)	25.23	4.03	0.34	0.52	0.34	0.26	0.43	24
14 SSR (excl. *Hse_214*)	24.78	2.68	0.34	0.52	0.35	0.26	0.44	24
14 SSR (excl. *Hse_210*)	24.74	2.65	0.33	0.52	0.37	0.27	0.45	24
12 SSR (excl. *Hse_186, Hse_426, Hse_471)*	25.68	3.78	0.35	0.47	0.26	0.16	0.36	24
11 SSR (excl. *Hse_214)*	25.69	3.53	0.35	0.45	0.23	0.12	0.33	24
11 SSR (excl. *Hse_210*)	25.64	3.36	0.33	0.44	0.24	0.13	0.35	24
10 SSR (excl. *Hse_210, Hse_214*)	25.65	3.04	0.33	0.41	0.19	0.08	0.31	22
**9 SSR (excl. *Hse_371*)**	26.21	3.18	0.35	0.40	0.14	0.02	0.26	16
8 SSR (excl. *Hse_489*)	26.11	3.16	0.35	0.39	0.12	-0.02	0.25	10

Table S5 Compilation of the mean genetic differentiation indices obtained for the different datasets. The data column indicates how many loci were still present in the dataset and which locus was excluded relative to the bigger dataset.

**Data**	**G'_{ST}**	**G'_{ST}_lower**	**G'_{ST}_upper**	**D_{EST}**	**D_{EST}_lower**	**D_{EST}_upper**
Total SSR data (15 loci)	0.118	0.100	0.135	0.060	0.047	0.0.69
14 SSR (excl. *Hse_186)*	0.112	0.091	0.130	0.050	0.035	0.058
14 SSR (excl. *Hse_426*)	0.110	0.092	0.127	0.050	0.036	0.058
14 SSR (excl. *Hse_471*)	0.110	0.098	0.125	0.051	0.045	0.059
14 SSR (excl. *Hse_214*)	0.105	0.085	0.121	0.050	0.035	0.058
14 SSR (excl. *Hse_210*)	0.112	0.093	0.129	0.050	0.035	0.058
12 SSR (excl. *Hse_186, Hse_426, Hse_471)*	0.094	0.079	0.108	0.030	0.023	0.036
11 SSR (excl. *Hse_214)*	0.076	0.063	0.090	0.020	0.014	0.026
11 SSR (excl. *Hse_210*)	0.086	0.072	0.100	0.020	0.014	0.026
10 SSR (excl. *Hse_210, Hse_214*)	0.066	0.051	0.081	0.018	0.012	0.024
**9 SSR (excl. *Hse_371*)**	0.057	0.045	0.070	0.014	0.009	0.019
8 SSR (excl. *Hse_489*)	0.048	0.035	0.061	0.010	0.005	0.016

S8. Population assignment test: Frequency based method

Table S6 Results of the population assignment test using the Frequency based method in GENECLASS2.

ID (Sex)	Sample location	Putative origin	p-value	Dispersal distance (km)
SGD02 (f)	SGD	SLD	0.010	8
SGD05 (f)	SGD	SLD	0.004	8
SLD11 (m)	SLD	BAL	0.001	175
SLD24 (m)	SLD	MZP	0.007	168
WEH07 (m)	WEH	MZP	0.000	181
BAL03 (f)	BAL	MZP	0.001	8
BAL28 (f)	BAL	HAG	0.001	14
BAL30 (m)	BAL	NIR	0.004	14
BBH07 (m)	BBH	KLB	0.000	33
BBH09 (m)	BBH	NIR	0.003	31
HAG02 (m)	HAG	MZP	0.001	20
HAG24 (m)	HAG	BAL	0.005	14
WEV14 (f)	WEV	NIR	0.000	21
WEV23 (m)	WEV	SCB	0.009	19
KDM26 (m)	KDM	ZBZ	0.008	13
KDM27 (f)	KDM	TLM	0.004	16
KLB22 (m)	KLB	KAH	0.003	89
MEH12 (m)	MEH	KDM	0.002	38
MEH20 (f)	MEH	HAG	0.004	36
SCB28 (f)	SCB	HHH	0.000	9
NIR10 (m)	NIR	TYZ	0.005	166
NIR12 (m)	NIR	TLM	0.000	26
NIR26 (m)	NIR	WEH	0.004	175
TLM01 (f)	TLM	MZP	0.001	22
TLM23 (f)	TLM	ZWI	0.001	142
TEW14 (m)	TEW	NIR	0.007	39
TEW18 (f)	TEW	NIR	0.004	39
ZBN08 (f)	ZBN	KAH	0.003	69
ZBN28 (m)	ZBN	TLM	0.002	6

The results of the population assignment test using the Frequency based method in GENECLASS2 identified 29 putative first-generation dispersers ($p <= 0.01$). Of these 29 dispersers, twelve (41%) were females. Putative dispersal events mainly occurred between inland populations (21 events, 72%), in which distances ranged between 8 and 89 km. We detected only two dispersal events between the same coastal populations (7%) with a dispersal distance of 8km. Three putative disperser originated in a coastal population and moved to an inland population (10%) (range: 142 – 175 km), and three putative disperser originated in an inland population and dispersed to a coastal population (10%) (range: 168 – 181 km).

S9. Genetic bottlenecks

Table S7 Results of the tests for detecting recent bottlenecks using the program BOTTLENECK v1.2.0.2 under the three models; Infinite Alleles Model (IAM), the intermediate Two-Phase Model (TPM) and the conservative Stepwise Mutation Model (SMM). Significant values ($p<0.05$) are indicated in bold.

Region	Pop	IAM	TPM	SMM
**Coastal**	SGD	0.2305	0.7695	0.9023
	SLD	**0.0195**	0.1484	0.2891
	TYZ	**0.0273**	0.3438	0.9453
	WEH	0.0977	0.5273	0.9727
	ZWI	**0.0098**	**0.0273**	0.7266
**Inland**	KAH	0.1484	0.4688	0.9727
	MZP	0.0820	0.3672	0.6738
	BAL	0.3203	0.7266	0.9805
	BBH	0.5000	0.8496	0.9990
	HAG	0.1914	0.6797	0.9805
	WEV	0.5273	0.9023	0.9981
	KDM	0.1875	0.7656	0.9961
	KLB	0.3203	0.6797	0.9727
	MEH	0.2344	0.7656	0.9727
	HHH	0.5273	0.9727	0.9961
	SCB	0.5273	0.9629	0.9961
	NIR	0.3262	0.6738	0.9815
	TLM	0.2481	0.8984	0.9971
	TEB	0.5273	0.7695	0.9727
	TEW	0.8750	0.9932	1.0000
	ZBN	0.7520	0.9971	1.0000
	ZBZ	0.9727	1.0000	1.0000
	MEH2001	0.1250	0.3711	0.9629
	TEB2001	0.5449	0.9756	0.9981

We tested for recent bottlenecks using the program BOTTLENECK v1.2.0.2 (8) which evaluates deviations of *He* from the values expected at mutation – drift equilibrium (*He* > *He eq*). We used the Wilcoxon's sign rank test (one-tailed test, *Ho*: no significant heterozygosity excess across all loci) which is the most powerful test and recommended for <20 loci (9). We used the results of the three mutational models, based on 10 000 simulation iterations: the Infinite Alleles Mode (IAM), the intermediate Two-Phase Model (TPM) and the more statistical conservative Stepwise Mutation Model (SMM). This is recommended when using microsatellite data since the true model of mutation for most loci is probably intermediate between the IAM and SMM (10). For the TPM, we used default values of 0.70 and 0.30 for the proportion of single-step mutations and variance, respectively. Significant values ($p < 0.05$) are indicated in bold.

S10. Estimation of effective population size (*Ne*)

<u>Ne estimation by using the LDNe method in NeEstimator v2.0</u>

Table S8 Results of the estimation of effective population sizes (Ne^) and the 95% Confidence Interval (95% CI) by using the LDNe method in NeEstimator v2.0.

Region	Location	Ne^	95% CI	
**Coastal**	SGD	36.4	7.8	Infinite
	SLD	103.7	12.3	Infinite
	TYZ	312.8	17.7	Infinite
	WEH	83.4	13.2	Infinite
	ZWI	119.3	10.1	Infinite
**Inland**	KAH	Infinite	42.8	Infinite
	MZP	60.8	7.8	Infinite
	BAL	Infinite	132.3	Infinite
	BBH	Infinite	11.5	Infinite
	HAG	Infinite	51.6	Infinite
	WEV	Infinite	28.5	Infinite
	KDM	Infinite	22.7	Infinite
	KLB	Infinite	14.2	Infinite
	MEH	Infinite	12.3	Infinite
	HHH	249.4	17.6	Infinite
	SCB	441.3	6.9	Infinite
	NIR	30.2	7.7	Infinite
	TLM	208.9	21.4	Infinite
	TEB	56.9	5.1	Infinite
	TEW	Infinite	37.6	Infinite
	ZBN	560.7	19.3	Infinite
	ZBZ	Infinite	22.4	Infinite
	MEH2001	373.8	9.0	Infinite
	TEB2001	27.6	3.0	Infinite

Results of the estimation of effective population sizes (Ne^) and the 95% Conficence Interval (95% CI) by using the LDNe method in NeEstimator v2.0 (11). The reported results are calculated with an allele frequency cut-off of 0.05 to avoid biased results due to the presence of rare alleles in the data, and under random mating model. We used the jack-knife option to obtain 95% confidence intervals. The LDNe method was not able to estimate an accurate effective population size (*Ne*) for any population, as the LDNe method produced an estimate with an infinite confidence interval for each population (12).

Ne estimation by using the sibship assignment method in the program Colony2

Table S9 Results of Ne estimation by using the sibship assignment method in the program Colony2. We report results under the random mating model (RM) and non-random mating model (N-RM) and their 95% confidence interval (95% CI) with the lower (L) and upper (U) limit. Mean_N are the mean number of genotyped individuals per population.

Region	Location	Mean_N	Ne (RM)	95% CI		Ne (N-RM)	95% CI	
				L	U		L	U
**Coast**	SGD	28.44	34	21	60	26	15	47
	SLD	29.00	32	19	59	17	10	36
	TYZ	29.44	26	15	46	18	10	38
	WEH	29.33	44	26	85	29	17	58
	ZWI	29.33	20	11	39	14	7	31
**Inland**	BAL	28.11	30	18	57	17	9	35
	BBH	26.00	33	19	60	23	13	43
	HAG	26.89	35	21	63	23	13	44
	KAH	27.11	32	19	57	20	11	42
	KDM	25.33	37	22	66	25	15	48
	KLB	21.78	36	20	72	23	12	47
	MEH	18.78	33	18	66	20	10	44
	HHH	27.44	36	22	64	31	18	56
	MZP	28.56	21	12	40	16	9	34
	NIR	24.44	25	14	46	16	9	35
	SCB	27.89	25	14	46	23	13	44
	TLM	27.22	32	18	58	20	11	41
	TEW	22.78	30	17	57	20	10	42
	TEB	19.89	32	18	64	23	12	46
	WEV	28.89	35	21	66	25	14	46
	ZBN	26.33	54	31	107	44	24	85
	ZBZ	23.89	39	23	84	35	19	69
	MEH 2001	23.44	28	16	57	19	10	40
	TEB 2001	16.89	31	16	69	29	15	61

We additionally used the sibship assignment method in the program Colony2 (13, 14) to estimate *Ne*. We used the following settings: random mating, female monogamy and male polygamy, no inbreeding, full-likelihood method and medium run length. For each population we ran a replica-run using different random number seeds to confirm the reliability of the analysis results.

S11. STRUCTURE and BAPS analysis

Bayesian analyses of population structure was performed with the programs STRUCTURE v2.3.4 (15) and BAPS v6 (16). The STRUCTURE clustering analysis was performed using an admixture model. We ran ten independent runs for each value of K (number of clusters) between 1 and 26 allowing for sub-structuring within sampling locations. The length of burn-in period was set at 100,000 followed by 200,000 Markov Chain Monte Carlo (MCMC) steps for each run. The optimal number of clusters (K) was determined using STRUCTURE HARVESTER (17). The BAPS clustering analyses were performed using the spatial model (with the geographic origin of the samples used as informative prior) for mixture clustering of individuals. We again ran ten independent runs with a fixed K-value, using upper bound K-values to 26. The most likely value of K was based on the maximum log-likelihood.

<u>Result of STRUCTURE analysis: STRUCTURE HARVESTER</u>

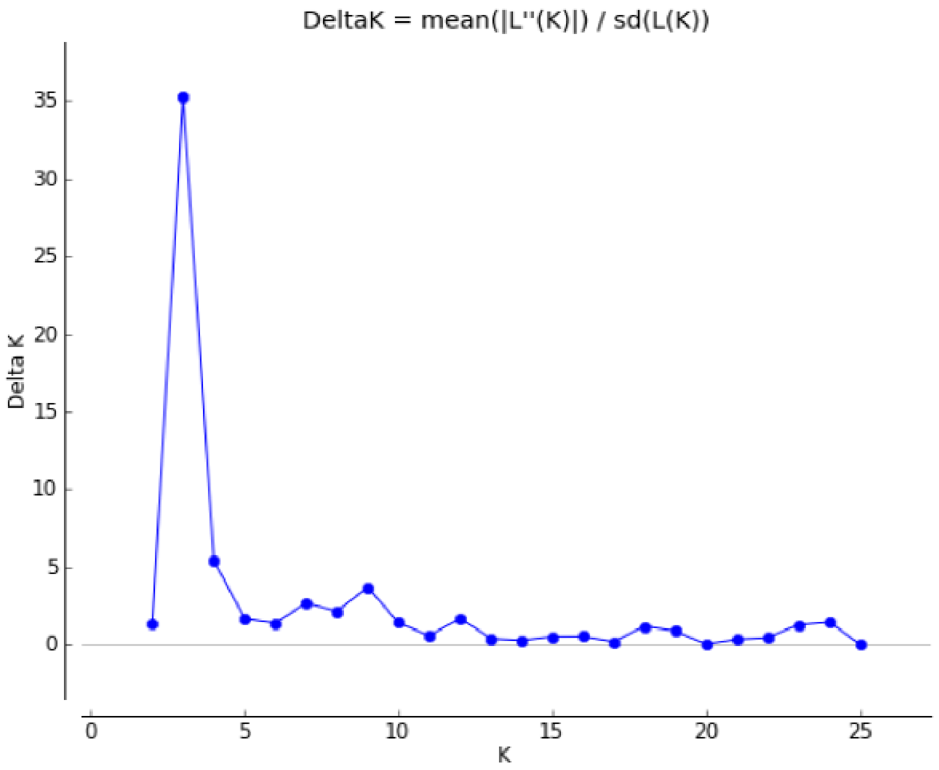

Figure S2 Delta K (ΔK) graph obtained by STRUCTURE HARVESTER showing an optimal number of 3 clusters (K).

Table S10 The Evanno table output obtained by STRUCTURE HARVESTER. Values for the optimal number of clusters (K = 3) are indicated in bold. With: the number of clusters (K) and the number of independent runs (Reps).

K	Reps	Mean LnP(K)	Stdev LnP(K)	Ln'(K)	|Ln"(K)|	Delta K
1	10	-9622.14	0.05	-	-	-
2	10	-9491.34	5.64	130.80	7.46	1.32
**3**	**10**	**-9368.00**	**4.72**	**123.34**	**166.36**	**35.26**
4	10	-9411.02	16.55	-43.02	89.42	5.40
5	10	-9364.62	26.56	46.40	44.43	1.67
6	10	-9362.65	14.81	1.97	20.65	1.39
7	10	-9340.03	32.13	22.62	85.92	2.67
8	10	-9403.33	50.48	-63.30	107.59	2.13
9	10	-9359.04	33.40	44.29	123.48	3.70
10	10	-9438.23	40.85	-79.19	57.38	1.40
11	10	-9574.80	173.45	-136.57	94.90	0.55
12	10	-9616.47	77.60	-41.67	131.53	1.70
13	10	-9789.67	248.67	-173.20	94.52	0.38
14	10	-9868.35	142.39	-78.68	37.80	0.27
15	10	-9984.83	120.27	-116.48	61.060	0.51
16	10	-10162.37	124.30	-177.54	64.28	0.52
17	10	-10275.63	217.29	-113.26	38.35	0.18
18	10	-10427.24	140.81	-151.61	165.74	1.18
19	10	-10413.11	177.08	14.13	151.65	0.86
20	10	-10550.63	186.95	-137.52	4.46	0.02
21	10	-10692.61	258.84	-141.98	85.20	0.33
22	10	-10749.39	242.82	-56.78	105.47	0.43
23	10	-10911.64	168.98	-162.25	214.67	1.27
24	10	-10859.22	178.38	52.42	258.46	1.45
25	10	-11065.26	210.53	-206.04	4.38	0.02
26	10	-11266.92	354.16	-201.66	-	-

Result of BAPS analysis

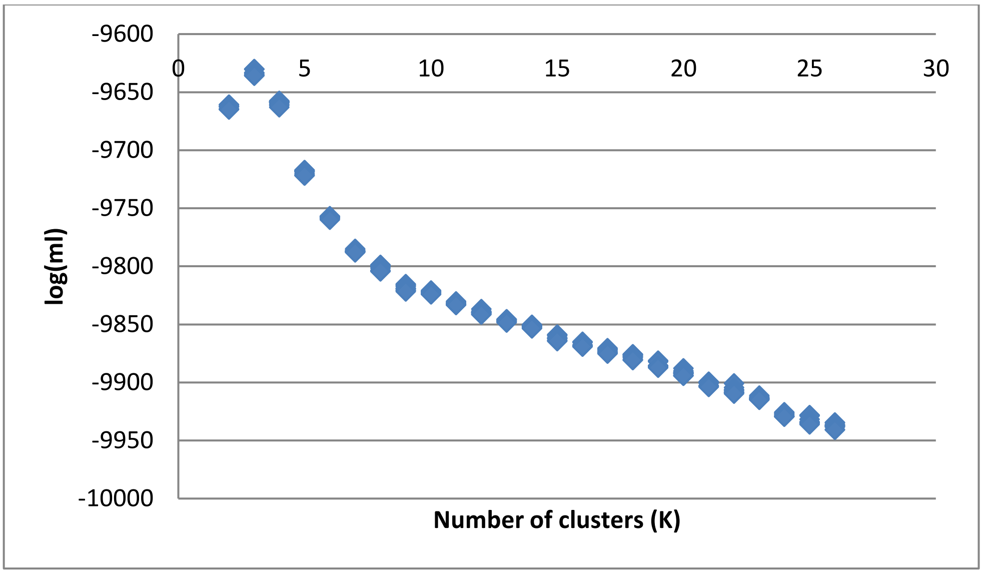

Figure S3 Results of the spatial clustering of individuals analyses with a maximum of 26 clusters (K) and ten independent runs for each. The optimal number of clusters with the highest marginal likelihood (log(ml)) was 3.

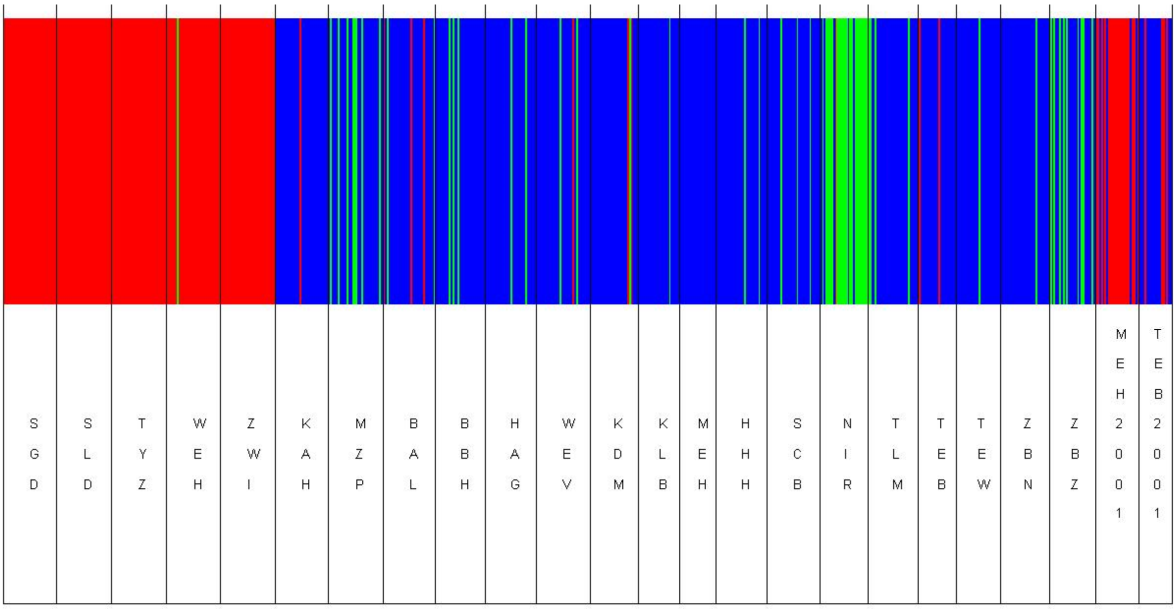

Figure S4 Estimated population structure for *Hipparchia semele* inferred by BAPS by using the spatial model, K=3. Each bar represents an individual, and the bar colours (red, blue, green) indicate the assigned cluster.

The coastal populations are all clustered together (red). All the inland heathland populations, except for NIR, are also assigned to one cluster (blue). The inland population NIR forms a separate cluster (green). Surprisingly, the 2001 population of MEH (inland) is clustered together with the coastal populations (red).

S12. Genetic diversity statistics

Table S11 Genetic diversity statistics of *Hipparchia semele* per population.

Region	Location	*N*	*A*	*Ae*	*Ar*	*Ap*	*Ho*	*He*	F_{IS}	F_{IS}_Low	F_{IS}_High
**Coastal**	SGD	28.444	3.222	1.851	2.880	0.010	0.353	0.386	0.086	-0.045	0.220
	SLD	29.000	2.778	1.841	2.600	0.000	0.270	0.373	0.278	0.159	0.388
	TYZ	29.444	3.333	1.992	2.990	0.000	0.356	0.421	0.155	0.023	0.281
	WEH	29.333	3.333	1.933	2.920	0.000	0.349	0.411	0.152	0.039	0.256
	ZWI	29.333	3.000	1.931	2.780	0.000	0.363	0.422	0.138	0.027	0.245
**Inland**	KAH	27.111	3.667	2.103	3.280	0.000	0.345	0.410	0.159	0.052	0.259
	MZP	28.556	3.333	2.064	2.980	0.000	0.375	0.422	0.111	0.010	0.197
	BAL	28.111	3.889	1.976	3.260	0.060	0.310	0.419	0.259	0.130	0.381
	BBH	26.000	4.000	2.091	3.380	0.000	0.356	0.413	0.139	0.024	0.257
	HAG	26.889	3.667	1.989	3.220	0.060	0.366	0.440	0.168	0.026	0.314
	WEV	28.889	4.222	2.097	3.510	0.020	0.374	0.428	0.127	0.015	0.240
	KDM	25.333	4.000	2.063	3.400	0.110	0.344	0.394	0.126	0.028	0.219
	KLB	21.778	3.444	1.980	3.070	0.070	0.321	0.399	0.194	0.096	0.289
	MEH	18.778	3.111	1.792	2.900	0.020	0.278	0.372	0.252	0.129	0.354
	HHH	27.444	3.889	2.021	3.260	0.070	0.381	0.407	0.065	-0.049	0.178
	SCB	27.889	3.556	1.829	3.040	0.010	0.350	0.350	-9e-04	-0.115	0.108
	NIR	24.444	3.556	1.989	3.170	0.000	0.356	0.442	0.195	0.021	0.359
	TLM	27.222	4.000	2.053	3.610	0.000	0.389	0.462	0.159	0.010	0.293
	TEB	19.889	3.222	1.847	2.910	0.040	0.319	0.372	0.142	-0.031	0.294
	TEW	22.778	4.000	1.909	3.450	0.070	0.324	0.381	0.150	0.019	0.271
	ZBN	26.333	4.333	2.042	3.650	0.110	0.395	0.418	0.056	-0.077	0.187
	ZBZ	23.889	4.111	1.916	3.350	0.090	0.377	0.398	0.054	-0.043	0.160
	MEH2001	23.444	2.889	1.787	2.730	0.000	0.319	0.369	0.136	-0.018	0.289
	TEB2001	16.889	3.444	1.850	3.170	0.100	0.356	0.396	0.101	-0.058	0.239

Genetic diversity statistics calculated with the programs GenAlEx v6.501 (18) and HP-Rare v1.0 (19) and the R package DiveRsity (20). Results are based on data of 9 microsatellite loci of 641 individuals of *Hipparchia semele* from 24 sampling locations in the coastal and inland heathland region of Flanders, Belgium. The mean number of individuals genotyped per locus (*N*); the mean number of observed alleles per locus (*A*); the effective number of alleles (*Ae*); the mean number of alleles per locus and corrected for sample size (based on a minimum of 13 individuals) (*Ar*); the number of private alleles corrected for sample sizes (based on a minimum of 13 individuals) (*Ap*); the observed heterozygosity (*Ho)*; the expected heterozygosity (*He*); the mean Wright's inbreeding coefficient per locus (F_{IS}); the lower limit of the 95% Confidence Interval of the corresponding F_{IS}-value (F_{IS}_*Low*);); the upper limit of the 95% Confidence Interval of the corresponding F_{IS}-value (F_{IS}_*High*).

S13. The effect of null alleles on the F_{IS}-values

We used the program INEst 2.2 (21) to get more insight on the effect of null alleles on the estimated inbreeding values. INEst 2.2 is a program that simultaneously calculates null allele frequencies and inbreeding values that are corrected for the estimated presence of null alleles. We used the IIM (Individual Inbreeding Model) model which is based on a Bayesian approach. We set the MCMC iterations to 200,000 cycles, the thinning parameter at 200 and the burn-in at 20,000 cycles.

Table S12 The frequency of null alleles calculated with the program INEst 2.2. Significant values are indicated in bold.

	Hse_454	Hse_269	Hse_350	Hse_343	Hse_007	Hse_376	Hse_229	Hse_369	Hse_489
**SGD**	/	0.0834	0.0116	0.1149	0.0430	0.1384	0.0299	0.0232	0.0268
**SLD**	/	0.0413	0.0575	**0.2100**	/	0.1739	0.0341	0.0908	0.2129
**TYZ**	/	0.0215	0.0167	0.0319	0.0220	/	0.0199	0.0297	0.0964
**WEH**	/	**0.1735**	0.0412	0.0869	**0.1931**	0.2250	0.0286	0.0780	0.0946
**ZWI**	/	0.0407	0.0271	0.0332	0.1130	0.0994	0.0207	0.0140	**0.2982**
**KAH**	/	0.0229	0.0118	0.0386	0.0558	/	0.0860	0.0290	0.1949
**MZP**	0.1283	0.0524	0.0086	0.0498	0.1351	**0.4935**	0.0194	0.0122	**0.2755**
**BAL**	/	0.0428	0.0322	0.1536	0.1640	0.3016	0.0412	0.0222	0.0806
**BBH**	0.0853	0.0328	0.0133	0.0356	0.0337	**0.3363**	0.0422	0.0153	**0.2684**
**HAG**	/	0.0539	0.0424	0.1201	**0.2152**	**0.4500**	0.0282	0.0154	0.1179
**WEV**	0.0429	0.0152	0.0351	0.0138	0.0317	/	0.0194	0.0233	0.0699
**KDM**	/	0.0126	0.0199	0.0642	0.0443	/	0.0342	0.0448	0.0753
**KLB**	0.0993	0.0248	0.0159	0.1759	0.0693	/	0.0647	0.0191	**0.2466**
**MEH**	/	0.0825	0.0277	0.1136	**0.3195**	/	0.0251	0.0688	**0.2934**
**HHH**	/	0.0131	0.0292	0.0218	0.1255	0.2194	0.0452	0.0141	0.0776
**SCB**	0.0244	0.0318	0.0071	0.0337	0.0268	/	0.0302	0.0161	0.1841
**NIR**	0.0937	0.0239	0.0272	0.0894	0.1388	0.2984	0.0569	0.0143	0.3327
**TLM**	0.0924	0.0388	0.0128	0.0543	0.1257	0.2476	0.0617	0.0202	0.1309
**TEB**	0.0352	0.0287	0.0176	0.0311	0.1430	/	0.1285	0.0169	0.0802
**TEW**	0.0452	0.0328	0.0306	0.1033	**0.2191**	0.2935	0.0530	0.0167	0.1517
**ZBN**	0.0617	0.0121	0.0206	0.0197	0.0214	0.2599	0.0232	0.103	0.0764
**ZBZ**	/	0.0181	0.0174	0.0255	0.1230	**0.4713**	0.0767	0.0188	0.1664
**MEH2001**	/	0.0151	0.0329	0.0837	0.1081	0.2161	0.0281	0.0786	0.0416
**TEB2001**	0.0481	0.0383	0.0294	0.0887	0.1293	0.3288	0.0207	0.0196	0.3148

We see that for the loci used in the data analyses, there are still 15 significant locus x population combinations (Table S12). This means that the presence of certain null alleles can have an influence on the estimation of F_{IS}-values. We therefore calculated F_{IS}-values corrected for these estimated frequencies of null alleles (Table S13).

Table S13 Comparison of the F_{IS}-values calculated with the R package DiveRsity (F_{IS}) and the program INEst 2.2(Corrected F_{IS}). Significant FIS-values estimated with DiveRsity are indicated in bold. For the corrected F_{IS}. the 95% highest posterior density interval (HPD 95%) is given. The best model indicates whether the presence of null alleles had an influence on the corrected F_{IS}-value (model nb) or not (model nfb).

Pop	F_{IS}	Corrected F_{IS}	HPD(95%)		Best model
			Low	High	
**SGD**	0.086	0.067	0.0000	0.1615	**nfb**
**SLD**	**0.278**	0.104	0.0000	0.2467	nb
**TYZ**	**0.155**	0.131	0.0000	0.2300	**nfb**
**WEH**	**0.152**	0.089	0.0000	0.2065	**nfb**
**ZWI**	**0.138**	0.039	0.0000	0.1138	nb
**KAH**	**0.159**	0.091	0.0000	0.2069	**nfb**
**MZP**	**0.111**	0.016	0.0000	0.0511	nb
**BAL**	**0.259**	0.137	0.0000	0.2849	**nfb**
**BBH**	**0.139**	0.039	0.0000	0.1215	nb
**HAG**	**0.168**	0.054	0.0000	0.1564	nb
**WEV**	**0.127**	0.118	0.0000	0.2138	**nfb**
**KDM**	**0.126**	0.096	0.0000	0.2239	nb
**KLB**	**0.194**	0.043	0.0000	0.1411	nb
**MEH**	**0.252**	0.052	0.0000	0.1688	nb
**HHH**	0.065	0.056	0.0000	0.1389	**nfb**
**SCB**	-0.001	0.056	0.0000	0.1389	nb
**NIR**	**0.195**	0.082	0.0000	0.2050	**nfb**
**TLM**	**0.159**	0.086	0.0000	0.2031	**nfb**
**TEB**	0.142	0.099	0.0000	0.2292	**nfb**
**TEW**	**0.150**	0.053	0.0000	0.1664	nb
**ZBN**	0.056	0.056	0.0000	0.1353	**nfb**
**ZBZ**	0.054	0.021	0.0000	0.0709	nb
**MEH2001**	0.136	0.087	0.0000	0.2145	**nfb**
**TEB2001**	0.101	0.042	0.0000	0.1315	nb

The corrected F_{IS}-values are overall (slightly) lower than the FIS-values calculated in the R package DiveRsity. Based on the highest posterior density intervals (HPD 95%), we can only say that there is weak evidence for the presence of inbreeding in the populations. As for a prior distribution, a beta distribution was chosen for inbreeding. Consequently, there is no possibility to directly verify whether inbreeding is larger from zero, because formally F cannot be equal zero under the beta prior (21).

To get more insight in whether the presence of null alleles or actual inbreeding is the most important parameter in calculating the corrected F_{IS}-value, we compared two models to each other. The first model, nfb, contained the following parameters: null alleles (n), inbreeding coefficients (f) and genotyping failures (b). In the second model, nb, the parameter of inbreeding coefficients was excluded. The model with the lowest DIC (Deviance Information Criterion) value was indicated as the best fitting model. If the nfb-model is selected as the best model, this means that the parameter 'inbreeding' was the most important factor in calculating the F_{IS}-values. If the nb-model is selected as the best model, this means that the F_{IS}-value is more influenced by the presence of null alleles (21).

For three of the coastal populations the nfb-model was the best fit. Two of these populations also showed significant inbreeding values calculated with DiveRsity. The nfb-model was also the best fit for nine of the inland populations, of which five populations showed significant inbreeding values calculated with DiveRsity (Table S13). Combining the methods, we thus found evidence of inbreeding with more certainty for seven of the 24 (30%) populations.

S14. Pairwise G'_{ST} values

Table S14 Pairwise G'_{ST} values; significant values are indicated in bold.

G'_{ST}	SGD	SLD	TYZ	WEH	ZWI	KAH	MZP	BAL	BBH	HAG	WEV	KDM	KLB	MEH	HHH	SCB	NIR	TLM	TEB	TEW	ZBN	ZBZ	MEH2001
**SGD**																							
**SLD**	0.036																						
**TYZ**	0.002	**0.048**																					
**WEH**	0.000	**0.050**	0.009																				
**ZWI**	**0.071**	**0.128**	**0.086**	**0.050**																			
**KAH**	**0.058**	**0.089**	0.036	0.036	**0.134**																		
**MZP**	**0.072**	**0.130**	**0.070**	**0.045**	**0.108**	**0.035**																	
**BAL**	0.026	**0.075**	0.018	0.003	**0.082**	0.012	0.010																
**BBH**	**0.044**	**0.079**	0.030	0.016	**0.119**	0.000	0.024	0.002															
**HAG**	**0.074**	**0.111**	**0.053**	**0.041**	**0.111**	0.031	**0.052**	0.015	0.031														
**WEV**	**0.045**	**0.090**	0.024	0.021	**0.115**	0.001	0.019	-0.004	-0.003	0.022													
**KDM**	**0.034**	**0.070**	0.027	0.018	**0.101**	0.018	0.023	-0.003	0.005	0.036	0.004												
**KLB**	**0.042**	**0.097**	0.028	0.014	**0.098**	0.012	0.014	-0.008	-0.010	0.029	-0.008	-0.004											
**MEH**	**0.083**	**0.133**	0.059	**0.065**	**0.160**	**0.060**	**0.080**	0.047	0.062	**0.068**	0.028	0.031	0.044										
**HHH**	0.034	**0.089**	0.022	0.010	**0.114**	0.006	0.018	-0.006	-0.002	0.024	-0.012	0.000	-0.005	0.030									
**SCB**	**0.093**	**0.145**	**0.103**	**0.060**	**0.129**	0.034	0.017	0.029	0.037	**0.051**	0.028	0.034	0.024	**0.095**	0.028								
**NIR**	**0.054**	**0.104**	0.044	0.037	**0.076**	0.051	0.028	0.034	0.023	**0.056**	0.037	0.034	0.018	**0.104**	0.041	**0.055**							
**TLM**	**0.101**	**0.151**	**0.083**	**0.047**	**0.096**	0.033	0.028	0.027	0.023	0.028	0.019	0.037	0.020	**0.081**	0.023	0.029	0.041						
**TEB**	0.040	**0.083**	0.029	0.016	**0.122**	-0.002	0.025	0.003	-0.002	0.031	-0.006	-0.002	-0.004	0.026	-0.004	0.024	0.038	0.026					
**TEW**	**0.076**	**0.114**	**0.079**	**0.053**	**0.145**	0.027	0.031	0.025	0.037	**0.045**	0.024	0.011	0.031	0.044	0.013	0.012	**0.069**	0.038	0.016				
**ZBN**	0.048	**0.091**	0.036	0.020	**0.105**	0.008	0.024	0.002	-0.001	0.027	-0.011	0.011	-0.004	0.040	0.001	0.031	0.033	0.019	-0.005	0.032			
**ZBZ**	**0.065**	**0.113**	**0.065**	0.035	**0.100**	0.038	0.018	0.014	0.009	**0.043**	0.012	0.028	0.006	**0.102**	0.018	0.023	0.019	0.026	**0.026**	0.055	0.006		
**MEH2001**	0.033	0.023	0.027	0.037	**0.109**	**0.053**	**0.097**	0.043	**0.067**	**0.087**	**0.057**	**0.049**	**0.067**	0.059	**0.058**	**0.114**	**0.104**	0.113	**0.042**	**0.073**	**0.060**	**0.111**	
**TEB2001**	0.041	**0.079**	0.030	0.011	**0.096**	0.008	0.032	0.008	-0.003	0.019	0.001	0.020	-0.002	0.063	0.009	0.026	0.014	0.016	-0.011	0.040	-0.006	0.004	0.056

S15. Isolation-by-distance analyses

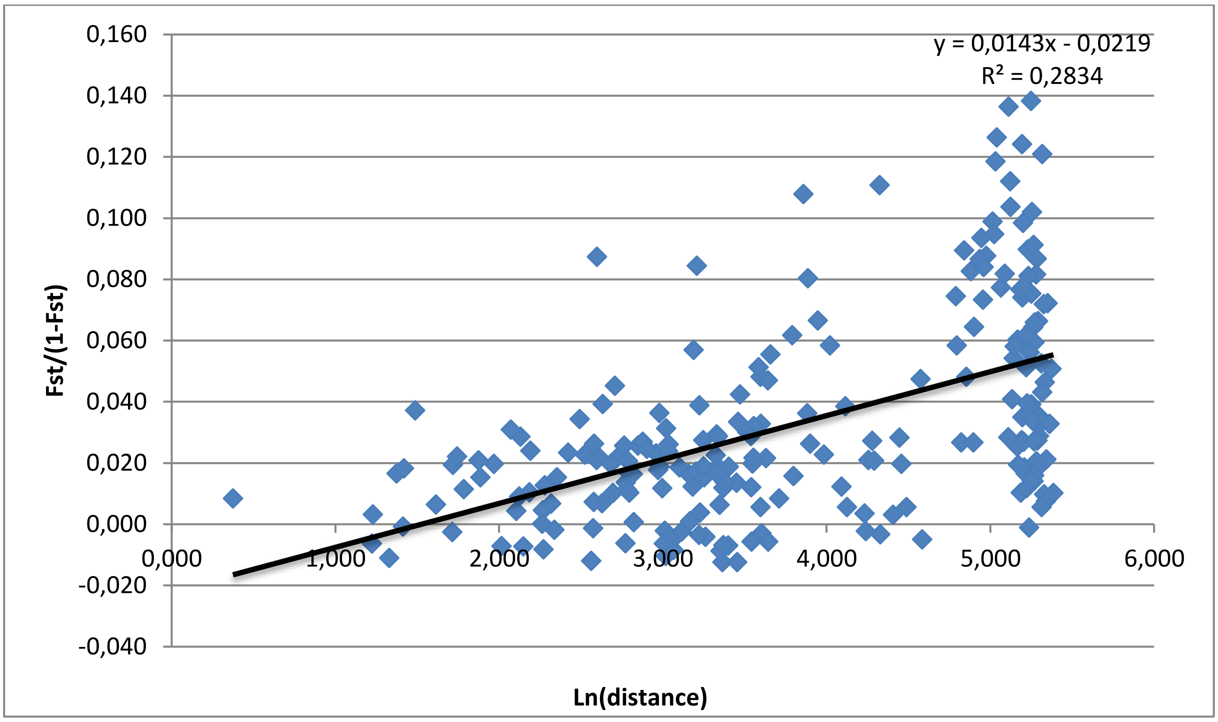

Figure S5 Global IBD-analysis with p-value = 0.00021 and R^2-value = 0.2834.

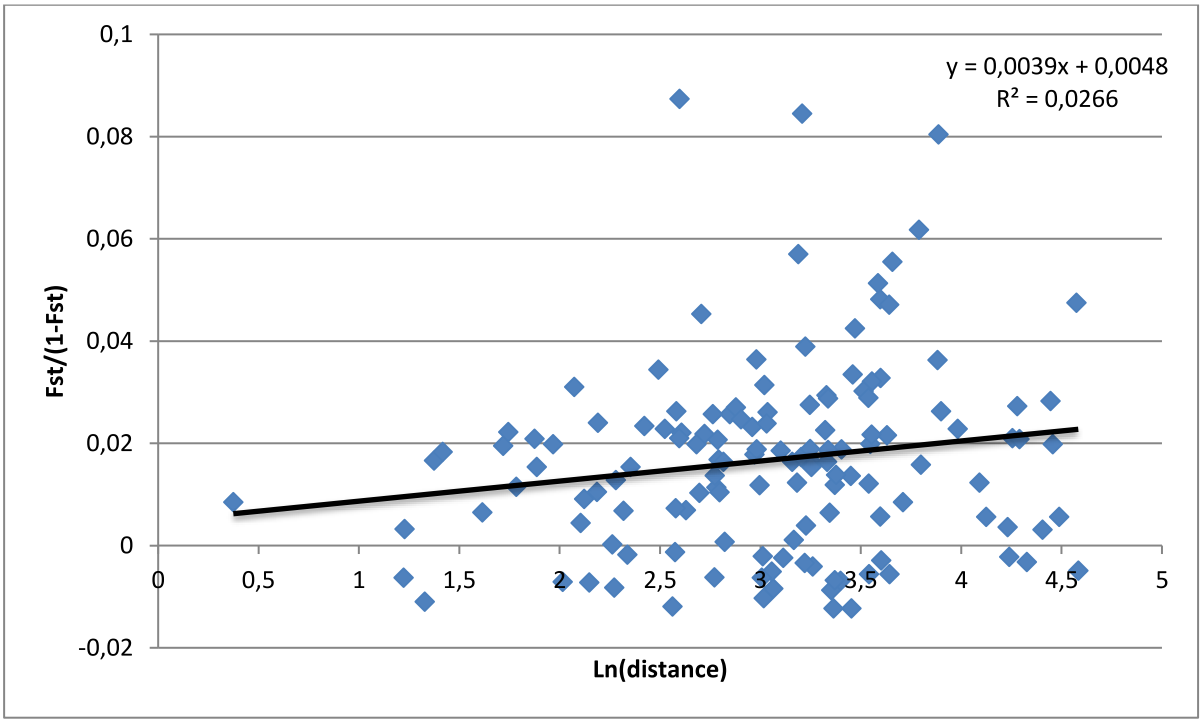

Figure S6 IBD-analysis of the inland region with p-value = 0.185 and R^2-value = 0.0266.

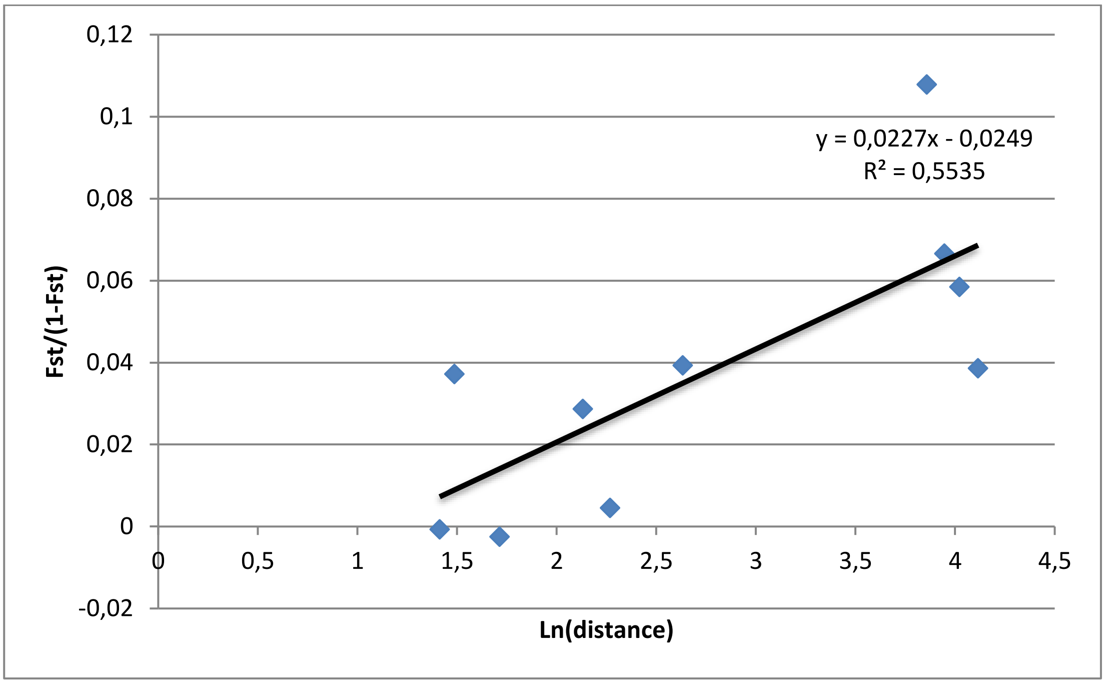

Figure S7 IBD-analysis of the coastal region with p-value = 0.087 and R^2-value = 0.5535.

S16. Levels of hierarchical structuring within populations, among populations and among regions estimated by analyses of molecular variance (AMOVA)

Table S15 Results of the AMOVA.

Source of Variation	Df	SS	MS	Estimated Variance	%	F-Stat	F-Value	P-value
**Among Regions**	1	33.01	33.01	0.061	3%	Frt	0.028	0.001
**Among Populations**	22	106.47	4.48	0.052	2%	Fsr	0.025	0.001
**Within Populations**	1258	2581.01	2.05	2.052	95%	Fst	0.052	0.001
**Total**	1281	2720.49		2.165	100%			

AMOVA performed with the program GenAlEx v6.501 (18) based on 9 microsatellite loci of 641 individuals of *Hipparchia semele* from 24 sampling locations nested within two regions: the coastal and inland heathland region of Flanders. Belgium. Significance was tested using 999 permutation.

S17. Plots of the Principal Component Analysis (PCoA)

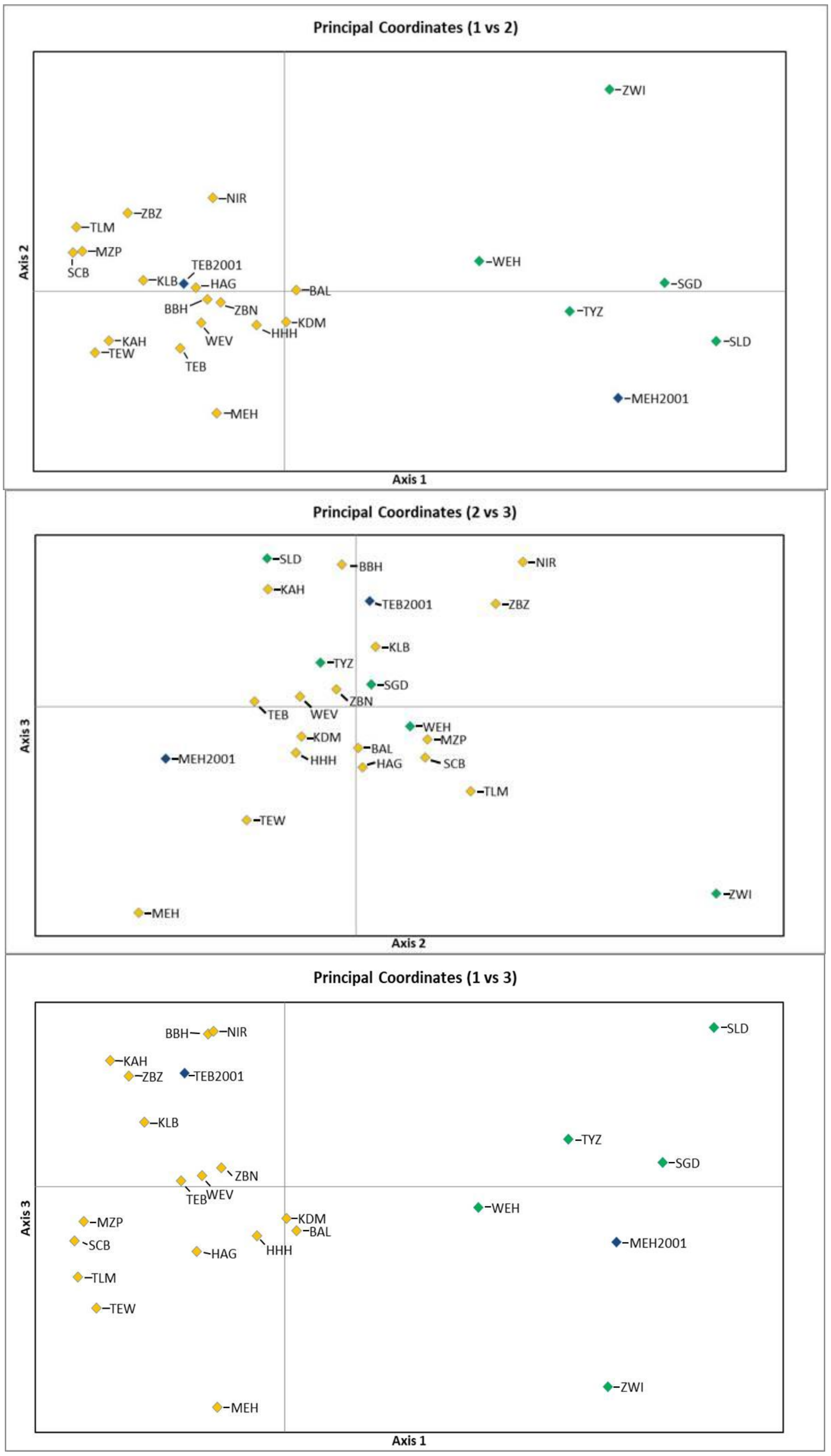

Figure S8 Plots of the Principal Component Analysis. The first three axes of the principal components represent 40.35% . 19.40% and 10.91% of the total variation in the data. First axis versus second axis (above). second axis versus third axis (middle). first axis versus third axis (bottom). Coastal populations are indicated in green. inland populations in yellow and the 2001 populations are indicated in dark blue.

S18. Detailed maps of the land-use in northern Belgium

The coastal region

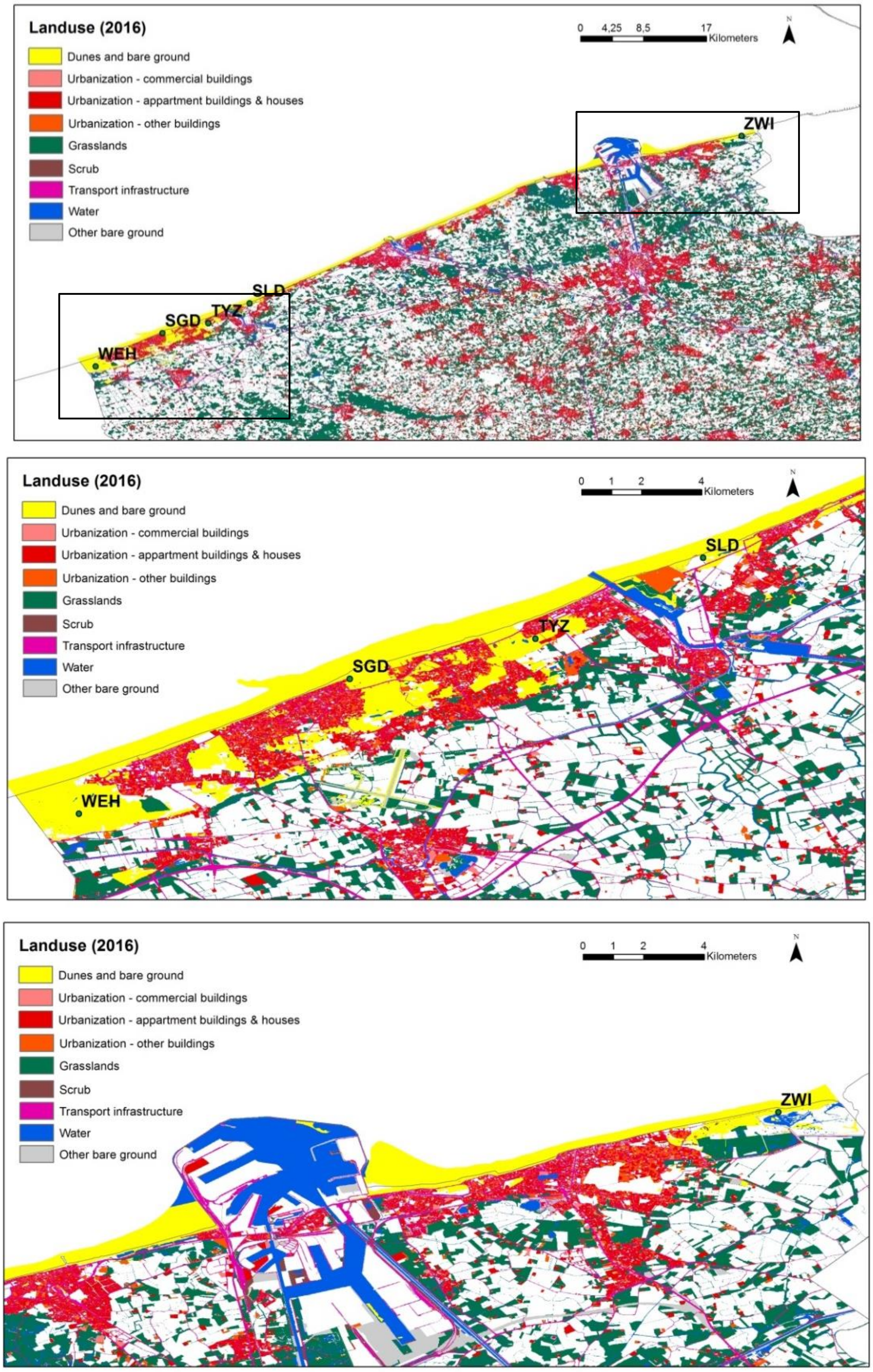

Figure S9 Detailed maps of the land-use in the coastal region of Belgium. Maps were created using: ArcGIS [GIS software]. Version 10.8. Redlands. CA: Environmental Systems Research Institute. Inc.. 2010.(www.esri.com). Source data landuse: Vlaamse Overheid – Departement Omgeving – Afdeling Vlaams Planbureau voor Omgeving. Landgebruik-Vlaanderen-2016 [GIS data]. Brussels. Belgium: Vlaamse Overheid. 2020. https://metadata.vlaanderen.be/srv/api/records/78e82101-8fbc-4a75-b649-b208df2b77be (visited: March 30. 2021).

The inland heathland region

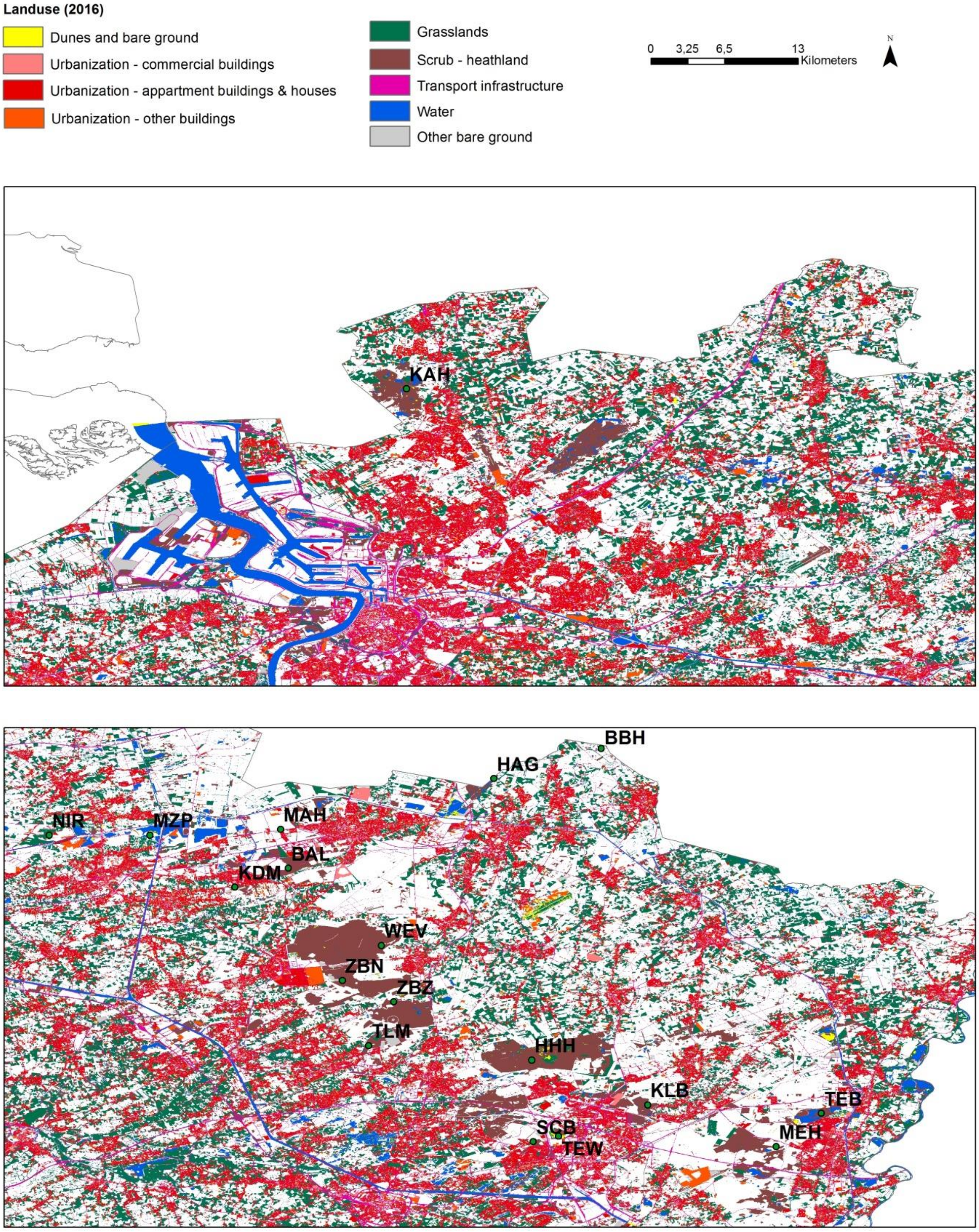

Figure S10 Detailed maps of the land-use in the inland heathland region in northern Belgium. Maps were created using: ArcGIS [GIS software]. Version 10.8. Redlands. CA: Environmental Systems Research Institute. Inc.. 2010.(www.esri.com). Source data landuse: Vlaamse Overheid – Departement Omgeving – Afdeling Vlaams Planbureau voor Omgeving. Landgebruik-Vlaanderen-2016 [GIS data]. Brussels. Belgium: Vlaamse Overheid. 2020. https://metadata.vlaanderen.be/srv/api/records/78e82101-8fbc-4a75-b649-b208df2b77be (visited: March 30. 2021).

References

1. Bolger AM, Lohse M, Usadel B. Trimmomatic: a flexible trimmer for Illumina sequence data. Bioinformatics. 2014;30(15):2114-20.
2. Koressaar T, Remm M. Enhancements and modifications of primer design program Primer3. Bioinformatics. 2007;23(10):1289-91.
3. Untergasser A, Cutcutache I, Koressaar T, Ye J, Faircloth BC, Remm M, et al. Primer3 - new capabilities and interfaces. Nucleic Acids Research. 2012;40(15):e115.
4. Schuelke M. An economic method for the fluorescent labeling of PCR fragments. Nature Biotechnology. 2000;18:233-4.
5. Holleley CE, Geerts PG. Multiplex Manager 1.0: a cross-platform computer program that plans and optimizes multiplex PCR. BioTechniques. 2009;46(7):511-7.
6. Dempster AP, Laird NM, Rubin DB. Maximum likelihood from incomplete data via the EM algorithm. Journal of the Royal Statistical Society. 1977;39(1):1-38.
7. Waples RS. Testing for Hardy-Weinberg proportions: have we lost the plot? J Hered. 2015;106(1):1-19.
8. Cornuet JM, Luikart G. Description and power analysis of two tests for detecting recent population bottlencks from allele frequency data. Genetics. 1996;144:2001-14.
9. Peery MZ, Kirby R, Reid BN, Stoelting R, Doucet-Beer E, Robinson S, et al. Reliability of genetic bottleneck tests for detecting recent population declines. Mol Ecol. 2012;21(14):3403-18.
10. Luikart G, Cornuet JM. Empirical evaluation of a test for identifying recently bottlenecked populations from allele frequency data. Conserv Biol. 1998;12(1):228-37.
11. Do C, Waples RS, Peel D, Macbeth GM, Tillett BJ, Ovenden JR. NeEstimator v2: re-implementation of software for the estimation of contemporary effective population size (Ne) from genetic data. Mol Ecol Resour. 2014;14(1):209-14.
12. Gilbert KJ, Whitlock MC. Evaluating methods for estimating local effective population size with and without migration. Evolution. 2015;69:2154-66.
13. Jones OR, Wang J. COLONY: a program for parentage and sibship inference from multilocus genotype data. Mol Ecol Resour. 2010;10(3):551-5.
14. Wang J. A new method for estimating effective population sizes from a single sample of multilocus genotypes. Mol Ecol. 2009;18(10):2148-64.
15. Pritchard JK, Stephens M, Donnelly P. Inference of population structure using multilocus genotype data. Genetics. 2000;155:945-59.
16. Corander J, Marttinen P. Bayesian identification of admixture events using multilocus molecular markers. Mol Ecol. 2006;15(10):2833-43.
17. Earl DA, von Holdt BM. STRUCTURE HARVESTER: a website and program for visualizing STRUCTURE output and implementing the Evanno method. Conservation Genetics Resources. 2012;4:359-61.
18. Peakall R, Smouse PE. GenAlEx 6.5: genetic analysis in Excel. Population genetic software for teaching and research--an update. Bioinformatics. 2012;28(19):2537-9.
19. Kalinowski ST. hp-rare 1.0: a computer program for performing rarefaction on measures of allelic richness. Molecular Ecology Notes. 2005;5(1):187-9.
20. Keenan K, McGinnity P, Cross TF, Crozier WW, Prodöhl PA, O'Hara RB. diveRsity: AnRpackage for the estimation and exploration of population genetics parameters and their associated errors. Methods in Ecology and Evolution. 2013;4(8):782-8.
21. Chybicki IJ, Burczyk J. Simultaneous estimation of null alleles and inbreeding coefficients. J Hered. 2009;100(1):106-13.
